# IRCMO: Biologically Inspired Dual-Population Coevolution via Immune Tolerance and Homeostatic Resource Allocation for Constrained Multi-Objective Optimization

**DOI:** 10.3390/biomimetics11090666

**Published:** 2026-09-16

**Authors:** Xiaoguo Chen, Yongchao Li, Xingsen Li, Yue Yang

**Affiliations:** 1 School of Information Engineering, Sanming University, Sanming 365004, China; 20180137@fjsmu.edu.cn; 2 School of Information and Electrical Engineering, Heilongjiang Bayi Agricultural University, Daqing 163319, China; 3 Institute of Extenics and Innovation Methods, Guangdong University of Technology, Guangzhou 510006, China; lixs@gdut.edu.cn; 4 College of Architecture and Civil Engineering, Sanming University, Sanming 365004, China; 20180136@fjsmu.edu.cn

**Keywords:** constrained multiobjective optimization, dual-population evolutionary algorithm, immune tolerance, niche exclusion, resource allocation, ecological complementarity

## Abstract

Constrained multiobjective optimization seeks Pareto optimal tradeoffs among conflicting objectives subject to prescribed constraints. Although the final solutions must be feasible, evolutionary variation can generate infeasible intermediate decision vectors during the search. One difficulty in this process is identifying mildly infeasible candidates that still provide valuable search directions, while another is distributing a fixed evaluation budget between cooperative populations whose contributions vary during evolution. To address these issues, this paper proposes IRCMO, a biologically inspired dual population constrained multiobjective evolutionary algorithm. The main population focuses on approximating the Pareto front formed by feasible solutions, while the auxiliary population preserves complementary search directions near constraint boundaries in the decision space. An Immune Tolerance and Niche Exclusion Selection strategy (ITNES) adaptively determines a tolerance boundary from the current feasibility status. It applies a squared response only to excess constraint violation and preserves sparse search directions in both the objective and decision spaces. This enables the auxiliary population to exploit mildly infeasible candidates without losing feasibility pressure, thereby improving convergence and front coverage under restrictive constraint structures. A Replicator Complementarity Homeostatic Resource Allocation strategy (RCHRA) evaluates offspring improvement and cross population complementarity. It assigns more offspring evaluations to the population making a stronger current contribution while maintaining a minimum resource share for both populations. This improves the utilization of the fixed evaluation budget and reduces persistent search imbalance between the two populations. Experiments on 47 benchmark problems and 12 real world CMOPs against seven representative algorithms show that IRCMO obtains the lowest overall average ranks for both IGD and HV. All pairwise Wilcoxon tests are significant at the 0.05 level.

## 1. Introduction

Real-world engineering optimization generally requires tradeoffs among multiple conflicting performance objectives while satisfying constraints imposed by physical laws, safety limits, resource capacities, and operational regulations. Representative applications include reactor network design [1], vehicle routing and scheduling [2], welded-beam and truss structural design [3,4], multiple-disk clutch brake design [5], and optimal pulse-width modulation for medium- and high-voltage power converters [6]. Recent multicriteria optimization studies have also addressed hospital case-mix planning [7] and transportation-network analysis [8], further illustrating the broad practical relevance of optimization under multiple criteria and operational restrictions. These tasks can be formulated within constrained multiobjective optimization or related multicriteria optimization frameworks. In CMOPs, the final solution set must satisfy all prescribed constraints while representing the tradeoffs among conflicting objectives. Evolutionary algorithms therefore need to identify feasible nondominated solutions while maintaining an adequate approximation of the Pareto front.

In general, a CMOP with *D* decision variables, *M* objective functions, and *L* constraints can be formulated as

(1) $$\begin{array}{cc} \min_{x}\;\; & F\left(x\right)={\left(f_{1}\left(x\right),\;f_{2}\left(x\right),\;\ldots ,\;f_{M}\left(x\right)\right)}^{T},\\ s.t.\;\; & g_{i}\left(x\right)\leq 0,\;i=1,\;\ldots ,\;p,\\ \; & h_{j}\left(x\right)=0,\;j=p+1,\;\ldots ,\;L,\\ \; & x\in S\subseteq R^{D}, \end{array}$$

where $x=(x_{1},\;x_{2},\;\ldots ,\;x_{D})$ is a *D*-dimensional decision vector, S denotes the decision space, and F(x) consists of *M* conflicting objective functions. The functions $g_{i}\left(x\right)$ and $h_{j}\left(x\right)$ denote inequality and equality constraints, respectively; the first *p* constraints are inequalities and the remaining L−p constraints are equalities. Evolutionary variation operators generate trial decision vectors throughout the search domain and do not, in general, preserve feasibility. Consequently, intermediate populations can contain candidates that violate one or more constraints even though only feasible solutions are admissible in the final solution set. To quantify the degree to which such an intermediate candidate violates the constraints, the violation of the *j*th constraint is defined as

(2) $$C_{j}\left(x\right)=\left\{\begin{array}{cc} \max\left\{0,\;g_{j}\left(x\right)\right\},\; & 1\leq j\leq p,\\ \max\left\{0,\;\left|h_{j}\left(x\right)\right|-\delta \right\},\; & p+1\leq j\leq L, \end{array}\right.$$

where δ is the numerical tolerance used for equality constraints to avoid an overly strict equality test under finite numerical precision. Based on this definition, the overall constraint violation (CV) [9] of an individual x is defined as

(3) $$CV\left(x\right)=\sum_{j=1}^{L}C_{j}\left(x\right).$$

Clearly, CV(x)≥0. A solution x is feasible if CV(x)=0, meaning that all constraints are satisfied; otherwise, x is infeasible, and a larger CV(x) generally indicates a more severe constraint violation. All feasible solutions jointly constitute the feasible region of a CMOP.

The feasible solutions that are not Pareto dominated by any other feasible solution constitute the constrained Pareto-optimal set, whose image in the objective space is referred to as the constrained Pareto front (CPF) [10]. If all constraints are ignored, the corresponding front is termed the unconstrained Pareto front (UPF). In addition to the general challenges of multiobjective optimization, such as representing increasingly complex Pareto sets and fronts as the number of objectives grows and efficiently solving computationally difficult subproblems, constraints introduce additional difficulties for evolutionary search. In particular, nonlinear constraints may divide the feasible set in the decision space into narrow, disconnected, or isolated components, while intervening infeasible regions in the decision space may hinder evolutionary transitions between promising feasible regions. When the CPF is substantially separated from the UPF, search driven primarily by objective improvement may repeatedly produce infeasible decision vectors with attractive objective values. Conversely, imposing strong feasibility pressure too early may eliminate mildly infeasible candidates near constraint boundaries before their search information can be exploited. Effective constrained evolutionary search therefore requires a suitable balance between exploiting informative infeasible candidates, guiding the population toward feasibility, and maintaining convergence and distribution quality.

Single-population evolutionary architectures combined with constraint-handling techniques remain a widely used approach to CMOPs, as represented by constraint domination in NSGA-II [11] and constrained decomposition methods such as MOEA/D-ACDP [12]. Multipopulation cooperation subsequently developed as a specialized research branch, with early collaborative formulations dating back to the early 2007. Within this branch, Liu et al. [13] formulated the problem as a two-population optimization problem, in which one population optimizes the constraints and the other optimizes the objective, with information and knowledge exchanged between them. CCMO employs constrained and unconstrained populations for cooperative search [14]. And AA-NSGA uses reference-line-guided archives to preserve informative infeasible trajectories [15]. More recent studies have further introduced adaptive cooperation, stage control, learning, and resource regulation [16,17,18,19,20].

Despite these advances, multipopulation CMOEAs still face two related issues. First, auxiliary search is often controlled by ignoring constraints, applying ε-relaxation, or using feasibility-based criteria. These mechanisms do not explicitly distinguish between infeasible candidates that merely have small violations and those that also contribute new objective- or decision-space directions. Second, the offspring budget is often fixed or adjusted according to short-term outcomes such as offspring survival. Such allocation may not reflect whether a population contributes search information that is complementary to that of the other population. These limitations indicate the need for a more selective assessment of auxiliary candidates and a resource-allocation mechanism that reflects both search effectiveness and cross-population complementarity. BiCo and C-TAEA in the experimental comparison represent methods with fixed cooperative resource allocation, while DRCMO represents constraint-relaxation-based search. C-TAEA also provides a representative case in which auxiliary diversity search is performed without explicit constraint pressure. Their comparative results provide direct experimental evidence supporting these observations.

Motivated by these observations, this paper proposes IRCMO, a dual-population cooperative constrained multiobjective evolutionary algorithm. The present study focuses on nonlinear CMOPs with low-to-moderate decision dimensionality. For auxiliary search, IRCMO retains mildly infeasible candidates only when their constraint violations remain acceptable and their objective- or decision-space information contributes distinct search directions. For resource allocation, the two populations are evaluated according to both offspring improvement and cross-population complementarity, and their offspring budgets are adjusted under a fixed total evaluation budget. Accordingly, IRCMO maintains a main population for feasible search and CPF approximation and an auxiliary population for complementary exploration, while shared offspring enable effective search information to be reused across the two populations. The main contributions are summarized as follows:

1. An Immune-Tolerance and Niche-Exclusion Selection strategy (ITNES) is proposed. Here, immune tolerance denotes an optimization analogy in which mildly infeasible candidates are conditionally retained rather than immediately rejected, whereas increasingly severe violations are progressively suppressed. ITNES combines this adaptive tolerance with joint objective–decision niche exclusion to preserve informative and differentiated search directions without turning the auxiliary search into unconstrained optimization.
2. A Replicator-Complementarity Homeostatic Resource Allocation strategy (RCHRA) is proposed. RCHRA evaluates the two populations according to both the reproductive quality of their offspring and the distinct search information contributed relative to the other population. These two signals are jointly used to adapt the offspring budgets under a fixed total evaluation budget, while a homeostatic regulation mechanism limits excessive resource concentration and maintains the participation of both populations throughout evolution.
3. Extensive experiments on 47 benchmark CMOPs and 12 real-world problems demonstrate the strong overall performance of IRCMO against seven representative algorithms in terms of both IGD and HV.

The remainder of this paper is organized as follows. Section 2 reviews gradual-evolution, multipopulation, multistage, and hybrid approaches to constrained multiobjective evolutionary optimization. Section 3 presents the overall framework and core mechanisms of IRCMO. Section 4 reports the experimental results on benchmark and real-world problems. Finally, Section 5 concludes the paper and discusses future research directions.

## 2. Related Work

A recurring issue in evolutionary approaches to CMOPs is the different search pressures induced by objective improvement and constraint satisfaction. When feasible regions in the decision space are narrow, disconnected, or difficult to reach, excessive feasibility pressure may restrict exploration, whereas insufficient constraint pressure may delay the search for feasible Pareto-optimal solutions. Existing CMOEAs address this issue from different perspectives and can be broadly organized into four paradigms, namely gradual-evolution methods, multipopulation cooperative methods, multistage methods, and hybrid methods. Table 1 provides a comparative overview of representative algorithms from these four categories in terms of constraint handling, resource allocation, computational complexity, population setting, and reported limitations. The detailed development of each paradigm is discussed below.

### 2.1. Gradual-Evolution Methods

Gradual-evolution methods regulate objective and constraint pressures within a continuous evolutionary process. MOEA/D-DAE temporarily relaxes constraints when search stagnation is detected through an improved ε mechanism [21]. DSPCMDE gradually shifts selection preference from objective-oriented to constraint-oriented search [22], while MOEA/D-2WA treats constraint violation as an additional objective and progressively adjusts infeasible weight vectors through a dynamic boundary [23]. CMOEA-SDE combines strict constrained dominance with constrained density estimation [24], and ACR adaptively regulates constraint violation using neighborhood information [25]. As summarized in Table 1, these methods generally rely on a single evolutionary structure with limited population-level resource coordination. Objective optimization and constraint handling therefore remain closely intertwined, which may restrict the simultaneous preservation of informative infeasible candidates and sufficient pressure toward feasible Pareto-optimal solutions.

### 2.2. Multipopulation Cooperative Methods

Multipopulation cooperative methods assign complementary search roles to different populations and exchange useful information during evolution. BiCo coordinates feasible- and infeasible-side searches through restricted mating [26]. MTCMO employs a relaxed auxiliary task with evolutionary multitasking [27], while DPACMO combines different constraint-handling strategies with population migration [28]. DPCPRA further adjusts offspring resources according to search feedback [29], and MCCMO controls multiple auxiliary populations through activation, dormancy, and merging mechanisms [30]. As summarized in Table 1, these methods differ not only in auxiliary constraint handling but also in resource coordination. However, auxiliary-population value is still often estimated from feasibility, relaxation, or immediate search outcomes, which may not fully reflect whether a population contributes nonreplaceable search information.

### 2.3. Multistage Methods

Multistage methods divide the evolutionary process into periods with different search purposes. PPS first ignores constraints and then introduces an improved ε mechanism [31]. C-TSEA uses unconstrained exploration followed by archive-assisted constrained search [32]. KTS adaptively switches between unconstrained and constrained surrogate-assisted search [33]. C3M progressively processes constraints according to their estimated priorities [34], while CMOES combines unconstrained search with an FNDS-based constraint-handling mechanism [35]. As summarized in Table 1, these methods reduce search conflicts through stage decomposition, but their performance remains sensitive to stage definitions and switching conditions. Premature or delayed switching may cause useful information loss or unnecessary evaluation consumption, while stage scheduling does not directly provide continuous resource redistribution between simultaneously useful search behaviors.

### 2.4. Hybrid Models

Hybrid methods combine multiple complementary mechanisms to improve adaptability to heterogeneous constraint structures. URCMO learns the relationship between the constrained and unconstrained Pareto fronts to guide subsequent dual-population search [36]. CMOQLMT uses Q-learning to adaptively select auxiliary tasks for knowledge transfer [37]. SC-EMT constructs auxiliary tasks from constraint subsets and coordinates them through two-stage evolution [38]. BPRRA combines dual-population and two-stage search with dynamic offspring-resource allocation [39], while DPTPEA adjusts interpopulation cooperation according to the feasibility state of the tractive population [40]. As summarized in Table 1, these methods provide greater adaptive flexibility, but auxiliary selection, task scheduling, and resource control are often driven by separate indicators, leaving individual search value and population-level contribution assessed in a fragmented manner.

Overall, the four paradigms summarized in Table 1 improve constrained multiobjective optimization through continuous pressure regulation, population-level division of labor, temporal decomposition, and hybrid adaptive coordination. However, two limitations remain relevant to dual-population search. First, the usefulness of infeasible candidates is still mainly assessed by constraint violation, relaxation, or objective quality, which does not explicitly indicate whether they provide distinct search directions. Second, resource allocation is often fixed or adjusted according to immediate outcomes such as offspring survival or feasibility, without considering whether one population contributes information that cannot be replaced by the other. These observations motivate IRCMO, in which ITNES evaluates auxiliary candidates through constraint tolerance and objective–decision diversity, while RCHRA allocates offspring resources according to reproductive success and cross-population complementarity.

## 3. Proposed Algorithm

### 3.1. Overall Framework of IRCMO

The overall procedure of IRCMO is summarized in Algorithm 1. Two populations of size *N* are initialized, and a fixed total offspring budget B=2N is specified. In each generation, binary tournament selection is independently applied to the main and auxiliary populations to construct mating pools, after which the standard genetic operator produces $O_{1}$ and $O_{2}$. RCHRA then evaluates the current parents and the actually generated offspring to determine the resource shares for the next generation. Offspring from both populations are simultaneously introduced into the candidate pools of the main and auxiliary populations, denoted by $Q_{1}$ and $Q_{2}$, allowing effective search directions discovered by either population to be reused by the other. It should be clarified that this information exchange is realized only through offspring sharing during environmental selection. The parent populations $P_{1}$ and $P_{2}$ are not directly merged or migrated. Instead, $O_{1}$ and $O_{2}$ are jointly supplied to both candidate pools, after which the main and auxiliary populations apply their own environmental selection mechanisms independently. Therefore, a solution generated from either population can contribute to the evolution of the other population only if it survives the corresponding environmental selection. The main population employs an SPEA2-style environmental selection based on constrained fitness and distance truncation [41], whereas the auxiliary population is updated using the proposed ITNES. After environmental selection, $N_{1}$ and $N_{2}$ are updated for the next generation. Upon termination, the main population provides the final approximation to the CPF. The environmental sizes of the two populations remain fixed at *N* throughout evolution. The quantities $N_{1}$ and $N_{2}$ refer only to the numbers of offspring generated by the two populations under the fixed total offspring budget B=2N, rather than to changes in the sizes of $P_{1}$ and $P_{2}$.

The two proposed mechanisms operate at complementary levels of dual-population search. ITNES regulates auxiliary-population selection through a feasibility-dependent tolerance, allowing the admissible degree of infeasibility to contract as feasible candidates become more abundant. The excess-violation response maintains a gradual feasibility pressure, while joint objective–decision niching preserves candidates that are diverse in both representation spaces. At the population level, RCHRA evaluates offspring improvement together with cross-population uniqueness to characterize the effective contribution of each population. Homeostatic regulation further moderates the resulting allocation and prevents temporary performance advantages from developing into persistent budget imbalance.

**Figure 1:**
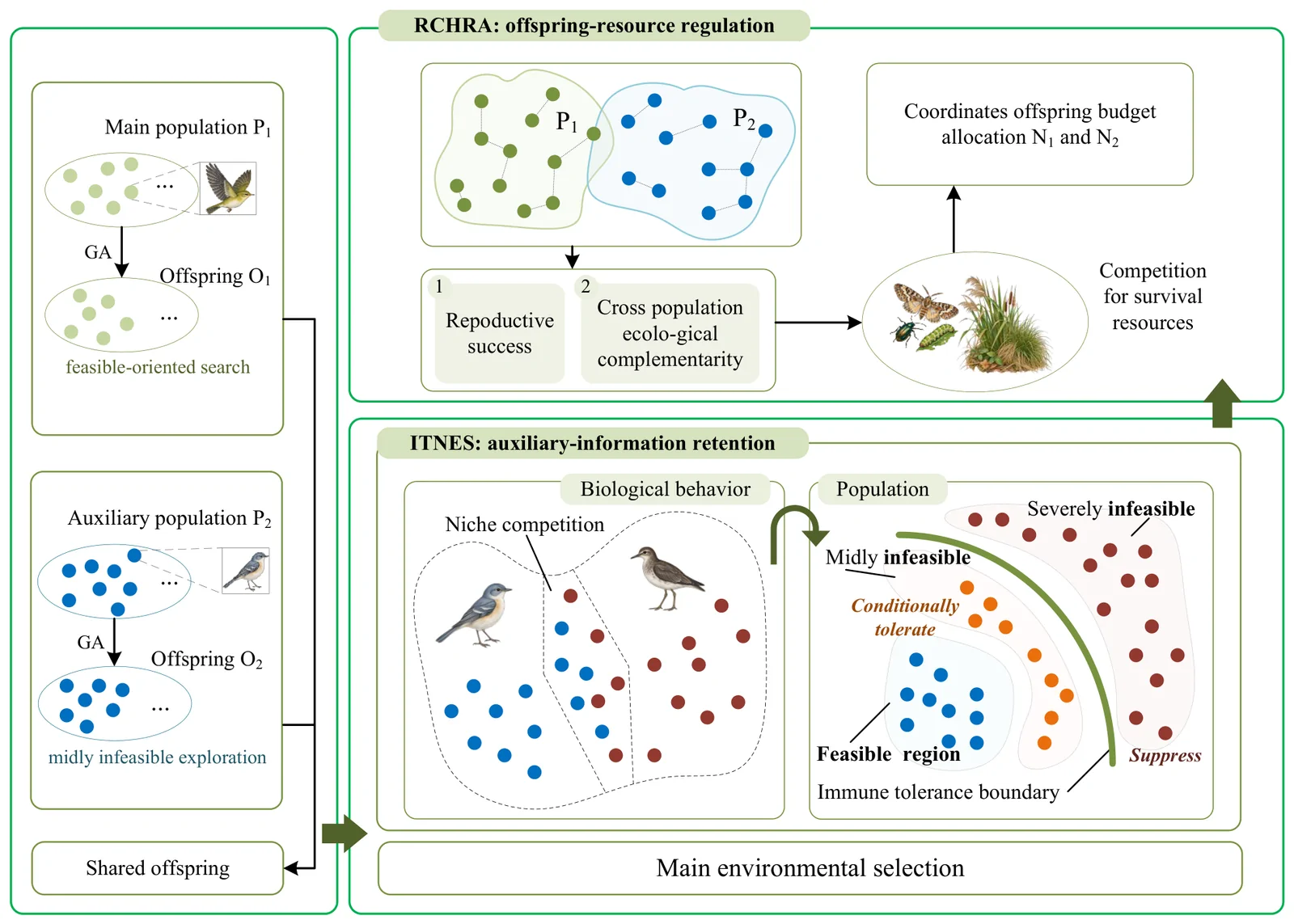
Conceptual mechanism diagram of IRCMO, showing the optimization meanings of immune tolerance and niche competition in ITNES and of reproductive success, ecological complementarity, and homeostatic offspring-budget regulation in RCHRA.

Figure 1 provides a conceptual map of the two mechanisms and their positions in the dual-population framework. At the individual level, ITNES interprets immune tolerance as conditional retention of mildly infeasible candidates and combines it with niche competition to preserve differentiated auxiliary information. At the population level, RCHRA interprets reproductive success as offspring improvement, ecological complementarity as nonredundant cross-population search information, and homeostasis as bounded feedback for offspring-budget regulation. The biological terms therefore serve as explicit design analogies for the corresponding optimization operations rather than as literal biological dynamics.
**Algorithm 1** Overall framework of IRCMO**Input:** 
Constrained multiobjective optimization problem P and population size *N***Output:** 
Main population $P_{1}$  1:Randomly initialize two populations $P_{1}$ and $P_{2}$ with $|P_{1}\left|=\right|P_{2}|=N$  2:Set the total offspring budget B←2N  3:$N_{1}\leftarrow 2\left\lfloor N/2\right\rfloor$, $N_{2}\leftarrow B-N_{1}$  4:**while** the termination criterion is not satisfied **do**  5:      Select $N_{1}$ parent indices $I_{1}$ from $P_{1}$ by binary tournament selection  6:      Select $N_{2}$ parent indices $I_{2}$ from $P_{2}$ by binary tournament selection  7:      $O_{1}\leftarrow \mathrm{OperatorGA}\left(P,P_{1}\left(I_{1}\right)\right)$  8:      $O_{2}\leftarrow \mathrm{OperatorGA}\left(P,P_{2}\left(I_{2}\right)\right)$  9:      $\left(N_{1}^{+},N_{2}^{+}\right)\leftarrow \mathrm{RCHRA}\left(P_{1},P_{2},O_{1},O_{2},B\right)$10:      $Q_{1}\leftarrow P_{1}\cup O_{1}\cup O_{2}$11:      $Q_{2}\leftarrow P_{2}\cup O_{1}\cup O_{2}$12:      $P_{1}\leftarrow \mathrm{MainSelection}\left(Q_{1},N\right)$13:      $P_{2}\leftarrow \mathrm{ITNES}\left(Q_{2},N\right)$14:      $N_{1}\leftarrow N_{1}^{+}$, $N_{2}\leftarrow N_{2}^{+}$15:**end while**16:**return** $P_{1}$

### 3.2. Immune-Tolerance and Niche-Exclusion Selection

The auxiliary population is intended to preserve search directions that may help cross constraint barriers, yet strict feasibility filtering may discard mildly infeasible candidates before their information is exploited, whereas completely ignoring constraints can drive the search away from the feasible target. ITNES therefore draws on the biological notion of immune tolerance, in which entities that do not require an immediate response are not indiscriminately rejected. Analogously, mildly infeasible candidates are conditionally retained according to the current feasibility status, while increasingly severe violations receive progressively stronger suppression. Different from auxiliary selection based on a fixed relaxation level or complete constraint neglect, ITNES determines the tolerance boundary directly from the feasibility composition of the current candidate set and applies additional pressure only to the violation exceeding this boundary. Moreover, diversity preservation is not performed solely in the objective space. The critical nondominated front is truncated according to a joint objective–decision distance so that solutions representing distinct search directions in both spaces can be retained. Therefore, adaptive tolerance, excess-violation regulation, and joint-space niche exclusion are integrated within the same environmental-selection process, enabling the auxiliary population to simultaneously regulate infeasibility and preserve structurally differentiated search information.

At generation *t*, the auxiliary environmental selection forms the candidate set $Q_{2}^{t}=P_{2}^{t}\cup O_{1}^{t}\cup O_{2}^{t}$ from $P_{2}^{t}$, $O_{1}^{t}$, and $O_{2}^{t}$. Let $N_{Q_{2}}=\left|Q_{2}^{t}\right|$, and let the target size of the auxiliary population be *N*. If $N_{Q_{2}}\leq N$, all candidates are retained directly. Otherwise, ITNES sequentially performs constraint-tolerance modeling, modified-objective ranking, and niche-based truncation.

First, the objective and decision matrices are normalized columnwise to obtain ${\hat{f}}_{i}$ and ${\hat{x}}_{i}$, respectively; if all values in a column are identical, that column is mapped to zero. Let $v_{i}\in \left[0,1\right]$ denote the normalized total constraint violation of individual *i*, and let $n_{f}$ denote the number of feasible candidates. The values $v_{i}$ are sorted in ascending order as $v_{\left(1\right)}\leq v_{\left(2\right)}\leq \cdots \leq v_{\left(N_{Q_{2}}\right)}$. ITNES determines the immune-tolerance order position according to the current proportion of infeasible individuals:

(4) $$q_{R}=\min\left\{N_{Q_{2}},N+\left\lceil \frac{\left(N_{Q_{2}}-N\right)\left(N_{Q_{2}}-n_{f}\right)}{N_{Q_{2}}}\right\rceil \right\}.$$

When the number of feasible solutions is small, $q_{R}$ approaches $N_{Q_{2}}$ and the tolerance region becomes relatively broad. As $n_{f}$ increases, $q_{R}$ gradually decreases and the tolerance region contracts accordingly. The tolerance threshold is therefore defined as $\tau_{R}=v_{\left(q_{R}\right)}$.

For individuals outside the tolerance boundary, ITNES modifies the normalized objectives using the squared excess-violation response in Equation (5). When $v_{i}\leq \tau_{R}$, competition is governed primarily by objective performance; when $v_{i}>\tau_{R}$, the same excess-violation term is added to all objectives. Because $v_{i}-\tau_{R}\in \left[0,1\right]$, the squared term suppresses mild excess violation more gently than a linear penalty, while still exerting strong pressure on severely infeasible individuals, thereby creating a continuous transition between exploration and constraint guidance.

(5) $${\bar{f}}_{im}={\hat{f}}_{im}+{\left[\max\left(0,v_{i}-\tau_{R}\right)\right]}^{2},\;m=1,\;\ldots ,\;M.$$

Nondominated sorting is applied once to the rows of the modified objective matrix $\bar{F}$ using standard Pareto dominance. Candidate *i* dominates candidate *j* when it is no worse in every modified objective and strictly better in at least one. In the implementation, this step uses the NDSort routine of PlatEMO [42]; constraint violation is not compared again because its excess effect has already been incorporated into $\bar{F}$ by Equation (5). Complete nondominated fronts preceding the critical front are directly admitted to the next generation, whereas the critical front is truncated through niche exclusion. To preserve both phenotypic differences in the objective space and structural, or genotypic, differences in the decision space, the joint niche distance is defined as

(6) $$d_{ij}=\sqrt{\frac{1}{2}\left(\frac{{∥{\hat{f}}_{i}-{\hat{f}}_{j}∥}_{2}^{2}}{M}+\frac{{∥{\hat{x}}_{i}-{\hat{x}}_{j}∥}_{2}^{2}}{D}\right)}.$$

where *M* and *D* denote the numbers of objectives and decision variables, respectively. Both distance components are normalized by their dimensionalities so that phenotypic differences in the objective space and structural differences in the decision space contribute on comparable scales to niche assessment.

Within the critical front, ITNES first reserves boundary-founder candidates for each modified objective direction. In case of ties, candidates are compared successively according to smaller constraint violation, smaller sum of modified objectives, and smaller original index. This procedure preferentially protects extreme solutions along different objective directions and prevents the truncation process from excessively shrinking the coverage of the auxiliary population.

Let *S* denote the set of currently retained individuals. The isolation of a candidate *i* relative to the occupied niches is defined as

(7) $$\rho_{i}\left(S\right)=\min_{j\in S}d_{ij}.$$

ITNES preferentially selects the candidate with the largest $\rho_{i}\left(S\right)$, i.e., the solution that is most isolated from the currently occupied niches. Ties in isolation are resolved successively using constraint violation, the sum of modified objectives, and the original index. Boundary-founder candidates are considered first, after which the remaining candidates in the critical front are selected until the auxiliary population reaches size *N*.

Algorithm 2 summarizes the complete ITNES procedure. Here, LexSelect(A,S) denotes lexicographic selection according to descending niche isolation, ascending constraint violation, ascending sum of modified objectives, and ascending original index. When the reference set *S* is empty, isolation is not evaluated and only the latter three criteria are used. Constraint violation therefore remains involved throughout the selection process through the modified objectives, boundary-candidate tie breaking, and niche-based secondary comparisons, allowing the auxiliary population to exploit informative infeasible candidates while maintaining pressure toward feasibility. Compared with conventional crowding-distance-based auxiliary selection, Algorithm 2 incurs additional computation from the joint-distance calculation and greedy max–min truncation. These operations are restricted to the critical nondominated front and are performed only when truncation is required. Nondominated sorting is executed once per environmental selection, and no additional objective or constraint evaluations are introduced. The resulting overhead supports the simultaneous assessment of constraint tolerance and objective–decision diversity.

### 3.3. Replicator-Complementarity Homeostatic Resource Allocation

The effectiveness of the two populations is not constant across evolutionary stages. One population may generate better offspring at a given stage, while the other may explore regions that are poorly represented by its counterpart. RCHRA is designed to distinguish these two contributions and translate them into offspring budgets. Replicator dynamics characterize reproductive success through the relative viability of offspring and parents. Complementarity measures the nonredundant search information contributed relative to the other population. Homeostatic regulation then moderates the allocation response to prevent temporary advantages from causing extreme or persistent resource imbalance. In this way, RCHRA integrates offspring quality and cross-population uniqueness under a fixed total evaluation budget.
**Algorithm 2** ITNES strategy**Input:** 
Auxiliary parent population $P_{2}^{t}$, offspring $O_{1}^{t}$ and $O_{2}^{t}$, and target size *N***Output:** 
Next-generation auxiliary population $P_{2}^{t+1}$  1:$Q_{2}^{t}\leftarrow P_{2}^{t}\cup O_{1}^{t}\cup O_{2}^{t}$  2:**if** $|Q_{2}^{t}|\leq N$ **then**  3:      **return** $Q_{2}^{t}$  4:**end if**  5:Normalize F and X columnwise to obtain $\hat{F}$ and $\hat{X}$  6:Count the number of feasible individuals $n_{f}$  7:Compute $q_{R}$ and $\tau_{R}$ according to Equation (4)  8:Construct $\bar{F}$ according to Equation (5)  9:$\left[r,r^{*}\right]\leftarrow \mathrm{NDSort}\left(\bar{F},N\right)$10:$S\leftarrow \{i|r_{i}<r^{*}\}$11:$A\leftarrow \{i|r_{i}=r^{*}\}$12:K←N−|S|13:**if** K>0 **then**14:      Compute the joint distance matrix D according to Equation (6)15:      $B_{f}\leftarrow ⌀$16:      **for** m=1 **to** *M* **do**17:            $A_{m}\leftarrow \arg\min_{i\in A}{\bar{f}}_{im}$18:            Select $b_{m}$ from $A_{m}$ lexicographically according to $\left(v_{i},\sum_{j=1}^{M}{\bar{f}}_{ij},i\right)$19:            $B_{f}\leftarrow B_{f}\cup \left\{b_{m}\right\}$20:      **end for**21:      **while** $|S|<N\wedge B_{f}\neq ⌀$ **do**22:            $i^{*}\leftarrow \mathrm{LexSelect}\left(B_{f},S\right)$23:            $S\leftarrow S\cup \{i^{*}\}$, $B_{f}\leftarrow B_{f}\setminus \left\{i^{*}\right\}$24:      **end while**25:      A←A∖S26:      **while** |S|<N∧A≠⌀ **do**27:            $i^{*}\leftarrow \mathrm{LexSelect}\left(A,S\right)$28:            $S\leftarrow S\cup \{i^{*}\}$, $A\leftarrow A\setminus \{i^{*}\}$29:      **end while**30:**end if**31:**if** |S|<N **then**32:      Sort unselected individuals by $\left(r_{i},v_{i},\sum_{m}{\bar{f}}_{im},i\right)$33:      Add individuals sequentially until |S|=N34:**end if**35:$P_{2}^{t+1}\leftarrow Q_{2}^{t}\left(S\right)$36:**return** $P_{2}^{t+1}$

Let the two parent populations at generation *t* be $P_{1}^{t}$ and $P_{2}^{t}$, with corresponding offspring $O_{1}^{t}$ and $O_{2}^{t}$. RCHRA merges the four sets into a common-garden candidate set $Q^{t}=P_{1}^{t}\cup P_{2}^{t}\cup O_{1}^{t}\cup O_{2}^{t}$, so that individuals from different sources are compared under the same objective and constraint scales. Let $N_{Q}=\left|Q^{t}\right|$, and let *M* and *D* denote the numbers of objectives and decision variables, respectively.

First, the constraint violation $CV_{i}$ of individual *i* is normalized to $v_{i}\in \left[0,1\right]$. Let $K=|P_{1}^{t}\left|+\right|P_{2}^{t}|$ denote the carrying size of the common garden, and let $n_{f}$ denote the number of feasible individuals in $Q^{t}$. After sorting $v_{i}$ in ascending order, Equation (8) determines the tolerance position *q* and tolerance boundary $\tau =v_{\left(q\right)}$, after which Equation (9) modifies the normalized objective ${\hat{f}}_{im}$. Individuals within the tolerance region compete mainly according to objective performance, whereas those beyond the boundary are suppressed in proportion to their excess violation. As the number of feasible individuals increases, the tolerance boundary automatically contracts.

(8) $$q=\min\{N_{Q},\max\left[1,\left\lceil K^{2}/\left(K+n_{f}\right)\right\rceil \right]\}$$

(9) $${\bar{f}}_{im}={\hat{f}}_{im}+\max\left(0,v_{i}-\tau \right),\;m=1,\;\ldots ,\;M.$$

The same standard Pareto nondominated sorting described in Section 3.2 is applied to the modified common-garden objective matrix, again using PlatEMO NDSort. The common-garden viability of individual *i* is defined as $a_{i}=1/r_{i}$, where $r_{i}$ is its nondomination rank. Equation (10) constructs the reproductive success $s_{k}$ of population *k* using the median viabilities of its offspring and parents. When $s_{k}>0.5$, the offspring generated by the population are, overall, more viable than their parents; otherwise, the corresponding evaluation budget has not yet been translated into sufficient reproductive gain. The median is used to reduce the influence of a small number of outlying individuals on population-level assessment.

(10) $$s_{k}=\frac{{\mathrm{med}}_{i\in O_{k}^{t}}a_{i}}{{\mathrm{med}}_{i\in O_{k}^{t}}a_{i}+{\mathrm{med}}_{i\in P_{k}^{t}}a_{i}},\;k\in \left\{1,2\right\}.$$

Although reproductive success reflects the capability of a population to generate high-quality offspring, it does not characterize the uniqueness of the search information carried by those offspring. Therefore, RCHRA reuses the joint objective–decision niche distance $d_{ij}$ defined in Equation (6) and computes cross-population ecological complementarity $c_{k}$ using Equation (11). Specifically, $c_{k}$ is the median of the nearest joint niche distances from offspring of population *k* to the union of the parents and offspring of the other population. It quantifies the discrepancy between the region explored by one population and the region already covered by the other. A larger $c_{k}$ indicates that the population reaches objective–decision regions that remain insufficiently explored by its counterpart, and hence provides a more unique and less replaceable search contribution.

(11) $$c_{k}=\underset{i\in O_{k}^{t}}{\mathrm{med}}\min_{j\in P_{3-k}^{t}\cup O_{3-k}^{t}}d_{ij}.$$

To reduce the direct influence of extreme reproductive-success or ecological-complementarity values on resource allocation, RCHRA employs mean-driven homeostatic normalization. For any nonnegative two-dimensional vector $z=(z_{1},z_{2})$, Equation (12) defines the normalization mapping $H_{k}\left(z\right)$, where $\bar{z}=\left(z_{1}+z_{2}\right)/2$. By introducing the current mean of the two populations, the transformation contracts the raw response moderately, preserving relative population advantages while maintaining a necessary level of resource competitiveness for the weaker population. On this basis, Equation (13) geometrically integrates the normalized reproductive success and ecological complementarity to obtain ecological fitness $e_{k}$. This construction requires a population to exhibit both good offspring quality and high cross-population complementary value, thereby preventing a population that is superior in only one criterion from receiving an excessively large resource share and balancing search effectiveness against information uniqueness.

(12) $$H_{k}\left(z\right)=\left(z_{k}+\bar{z}\right)/\sum_{j=1}^{2}\left(z_{j}+\bar{z}\right).$$

(13) $$e_{k}=\sqrt{H_{k}\left(s\right)H_{k}\left(c\right)},\;s=\left(s_{1},s_{2}\right),\;c=\left(c_{1},c_{2}\right).$$

Let the current resource share be $p_{k}^{t}=|O_{k}^{t}\left|/(\right|O_{1}^{t}\left|+\right|O_{2}^{t}\left|\right)$; if offspring have not yet been generated, the parent-size ratio is used for initialization. To prevent historical advantages from accumulating across generations through direct use of $p_{k}^{t}e_{k}$, RCHRA first contracts the historical share using ${\tilde{p}}_{k}^{t}=H_{k}\left(p^{t}\right)$ and then takes the geometric midpoint between the homeostatically adjusted historical share and current ecological fitness in Equation (14), producing the continuous resource share $p_{k}^{t+1}$ for the next generation. Finally, the continuous shares are projected onto integer budgets compatible with paired genetic operators. Let T=B/2 and $T_{\min}=\max\left\{1,\left\lceil T/4\right\rceil \right\}$. Equation (15) determines the number of reproductive pairs $T_{1}$ for the first population, and Equation (16) gives $N_{1}$ and $N_{2}$. This projection guarantees $N_{1}+N_{2}=B$ and keeps both budgets positive and even, while structurally reserving approximately one quarter of the total budget for each population to reduce the risks of resource monopolization and population degeneration.

(14) $$p_{k}^{t+1}=\frac{\sqrt{{\tilde{p}}_{k}^{t}e_{k}}}{\sum_{j=1}^{2}\sqrt{{\tilde{p}}_{j}^{t}e_{j}}},\;k\in \left\{1,2\right\}.$$

(15) $$T_{1}=\max\left\{T_{\min},\min\left\{T-T_{\min},\mathrm{round}\left(Tp_{1}^{t+1}\right)\right\}\right\}.$$

(16) $$N_{1}=2T_{1},\;N_{2}=B-N_{1}.$$

### 3.4. Complexity Analysis

Let the size of each population be *N*, and let *M*, *D*, and *L* denote the numbers of objectives, decision variables, and constraints, respectively. RCHRA has a time complexity of $O(\left(M+D\right)N^{2}+NL)$ and a space complexity of $O(N^{2})$. For ITNES, let $N_{c}$ denote the size of the critical nondominated front. Its greedy max–min truncation requires at most $O(N_{c}^{3})$ time, where $N_{c}\leq N$. Therefore, the $O(N^{3})$ term represents a strict worst-case bound. It is reached only when the critical front grows to the population scale and requires extensive truncation. Such a case does not occur in every generation. The SPEA2-style main-population selection has a worst-case complexity of $O(N^{3}\log N+MN^{2}+NL)$. Hence, the overall worst-case time complexity of IRCMO is $O(N^{3}\log N+\left(M+D\right)N^{2}+NL)$. Its space complexity is $O(N^{2})$.

## 4. Experiments

### 4.1. Experimental Settings

To comprehensively evaluate the feasibility, convergence, and distribution performance of IRCMO, four widely used benchmark suites are employed: CF [43], DAS-CMOP [44], LIR-CMOP [45], and MW [46], comprising 47 problems in total. Table 2 summarizes the principal properties of the four benchmark suites. All 47 problems use continuous real-valued decision variables and are nonlinear CMOPs, covering two- and three-objective settings with decision-space dimensionalities ranging from 10 to 30. The suites further include heterogeneous characteristics such as nonlinear variable linkages, multimodality, disconnected or narrow feasible regions, and nonlinear constraint boundaries, providing diverse constrained search scenarios for evaluating IRCMO.

The comparison algorithms are BiCo [26], C3M [34], ToP, APSEA [47], C-TAEA [48], DRCMO [49], and MGCMOEA [50]. These algorithms cover representative technical paradigms including multipopulation search, multistage search, hybrid models, and dynamic resource allocation, providing a comprehensive comparative baseline.

All algorithms use the parameter settings recommended in their original publications and are independently run 30 times on each test problem. Statistical significance is assessed using the two-sided Wilcoxon signed-rank test at a significance level of α=0.05. Pairwise tests are conducted between IRCMO and each comparison algorithm based on their results over all test problems.The base population size is set to N=100 [27], and the maximum number of function evaluations is 100,000 (MaxFES). All experiments are conducted on the PlatEMO platform [42] to ensure consistency in the implementation environment and evaluation budget.

Performance is evaluated using Inverted Generational Distance (IGD) [51], Hypervolume (HV) [52], and Feasible Solutions Ratio (FSR) [53]. A smaller IGD indicates better convergence and coverage with respect to the reference front, while a larger HV indicates better convergence and distribution. FSR reflects the stability with which an algorithm obtains feasible solutions. For IGD, the reference set is generated by the corresponding benchmark problem in PlatEMO using 10,000 reference samples. For HV, the objective values are normalized according to the reference front with a 10% extension, and the reference point is set to the unit vector in the normalized objective space.

### 4.2. Results on Benchmark Problems

Table 3 and Table 4 summarize the IGD and HV results of the eight algorithms on the 47 benchmark problems. Each entry reports the mean and standard deviation over 30 independent runs. When an algorithm cannot reliably obtain feasible solutions, the corresponding metric is reported as NaN, with FSR given in parentheses. The symbols “+”, “−”, and “=” indicate that the comparison algorithm is significantly worse than, significantly better than, or statistically indistinguishable from IRCMO, respectively. Boldface identifies the best mean value on each problem.

On the CF suite, IRCMO performs best on most problems from CF3 to CF10, while C3M and MGCMOEA are more competitive on CF1 and CF2. Several algorithms fail to obtain stable feasible solutions on CF8 and CF10, whereas IRCMO remains effective. This result indicates that IRCMO maintains good robustness across different constraint structures in the CF suite.

On the DAS-CMOP suite, IRCMO shows strong performance on DAS-CMOP3–DAS-CMOP8, while C3M performs better on several other instances. Since DAS-CMOP separately controls feasibility, convergence, and diversity difficulties, the results suggest that IRCMO adapts well to different combinations of these challenges. Nevertheless, stage-wise constraint handling remains advantageous on some specific problems.

On LIR-CMOP, IRCMO achieves lower IGD on most instances and competitive HV values on several problems. Its performance is particularly strong on problems containing large infeasible regions and disconnected feasible regions. However, C3M, C-TAEA, and DRCMO remain more competitive on several specific instances, showing that strongly directed or stage-wise search can still be beneficial for particular constraint structures.

The clearest overall advantage is observed on the MW suite, where IRCMO obtains the best mean IGD and HV values on all 14 problems. It also maintains feasible approximation sets on several difficult instances for which some comparison algorithms return NaN or very low HV values. Overall, the results support the effectiveness of the proposed auxiliary-selection and adaptive resource-allocation mechanisms, while also showing that the relative advantage depends on the structure of the constrained problem.

### 4.3. Results on Real-World Problems

Table 5 lists the 12 real-world CMOPs, covering structural design, mechanical design, crash-energy management for high-speed trains, pooling, and process synthesis. The IGD results in Table 6 show that IRCMO achieves strong convergence on most engineering problems, with particularly notable performance on RWMOP1, RWMOP4, RWMOP6, and RWMOP8. This suggests that ITNES preserves useful search information across constrained regions, while RCHRA improves the utilization of the evaluation budget. RWMOP5, RWMOP7, and RWMOP9 yield nearly identical results for most algorithms, indicating limited discriminatory power on these instances. IRCMO therefore shows comparable performance on these cases rather than a clear advantage. C3M attains a better IGD on RWMOP3. On RWMOP10, IRCMO fails to obtain a feasible solution while several comparison algorithms reach the feasible region, indicating that extremely narrow or structurally unusual feasible regions remain challenging. The HV results in Table 7 further show competitive overall performance, although the relative advantage depends on problem structure. Taken together, IRCMO maintains stable convergence and competitive distribution quality across the real-world problems, while showing clearer advantages on several more discriminative instances. Feasibility recovery for particularly difficult regions remains an important direction for further improvement.

### 4.4. Diversity Analysis

**Figure 2:**
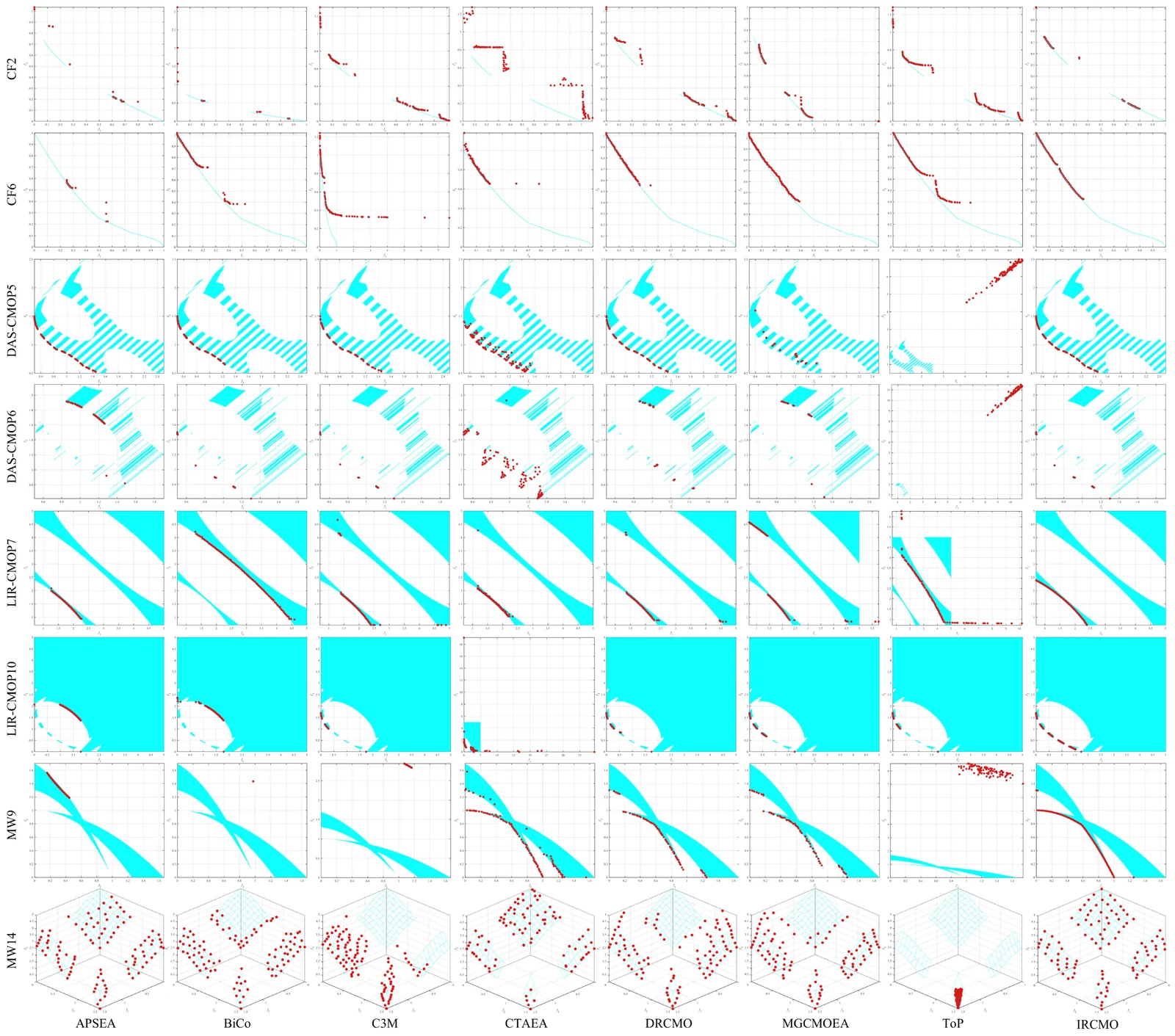
Final Final population distributions of the eight algorithms on representative benchmark problems. The blue area denotes the feasible region, and the red dots represent the population individuals.

Figure 2 presents the final population distributions on eight representative problems. Overall, IRCMO follows the CPF more continuously and maintains more complete coverage on disconnected fronts and narrow feasible regions. It covers the major effective front segments on both CF2 and CF6, exhibits less pronounced deviation or local clustering on DAS-CMOP5 and DAS-CMOP6, forms continuous solution sets despite the large infeasible regions of LIR-CMOP7 and LIR-CMOP10, and covers multiple disconnected regions on MW9 and the three-objective MW14. This behavior is consistent with the role of ITNES: immune tolerance prevents potentially useful infeasible directions from being discarded prematurely, while joint objective–decision niche exclusion further suppresses local crowding, allowing differentiated search information to continuously replenish the main population through shared offspring.

### 4.5. Convergence Analysis

**Figure 3:**
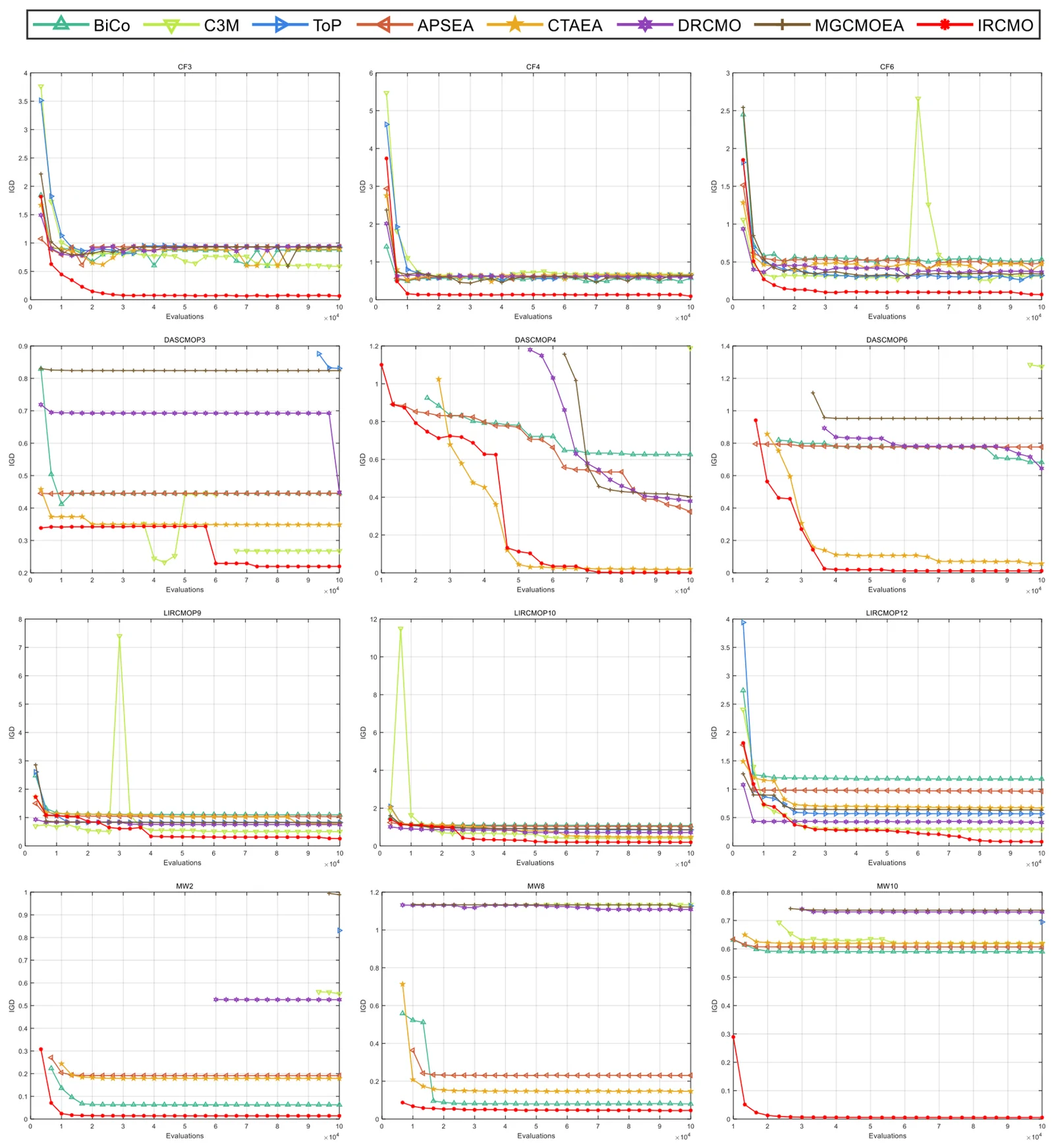
IGD convergence curves of the eight algorithms on representative benchmark problems.

Figure 3 shows the IGD convergence curves on 12 representative problems. IRCMO reduces IGD rapidly on CF3, CF4, CF6, MW2, MW8, and MW10 and maintains the lowest or near-lowest values during the later stages. On more difficult problems such as DAS-CMOP4, DAS-CMOP6, and LIR-CMOP9–LIR-CMOP12, IRCMO does not always lead in the early stage, but it continues to improve after effective feasible directions are identified, whereas several comparison algorithms reach plateaus earlier. In particular, IRCMO continues to decrease in the middle and later stages of DAS-CMOP4 and DAS-CMOP6, indicating that its search directions are not prematurely fixed by early resource allocation. ITNES continuously preserves entries to boundary and sparse regions for subsequent barrier crossing, while RCHRA readjusts offspring budgets according to current reproductive gain and complementarity; together, they reduce the risk of long-term stagnation.

### 4.6. Actual Runtime Analysis

The theoretical complexity analysis characterizes the asymptotic computational cost of IRCMO, but it does not directly reflect the actual execution time because practical runtime is also affected by implementation details, population operations, and problem evaluation costs. Therefore, the actual running times of IRCMO and the seven comparison algorithms were further recorded on the five problem sets. Table 8 reports the mean running time in seconds, with the corresponding standard deviation given in parentheses.

The results show that the additional operations introduced by ITNES and RCHRA do not lead to prohibitive computational overhead in practice. On DAS-CMOP, its runtime is almost identical to that of C3M and is substantially lower than those of ToP, C-TAEA, DRCMO, and MGCMOEA. On CF, LIR-CMOP, and MW, IRCMO is slower than several structurally simpler algorithms, such as BiCo and C3M, but remains considerably faster than C-TAEA, DRCMO, and MGCMOEA. On the real-world problems, IRCMO achieves the second-lowest running time among the eight algorithms. The relatively small standard deviations across all five problem sets also indicate stable computational behavior among independent runs. Overall, the additional computation required for adaptive tolerance estimation, joint objective–decision niche evaluation, and complementarity-based resource allocation introduces a moderate rather than excessive runtime cost. Although the worst-case complexity contains a cubic component associated with environmental selection, the measured results show that this theoretical upper bound does not translate into excessive runtime under the experimental settings considered in this study.

### 4.7. Statistical Analysis

**Figure 4:**
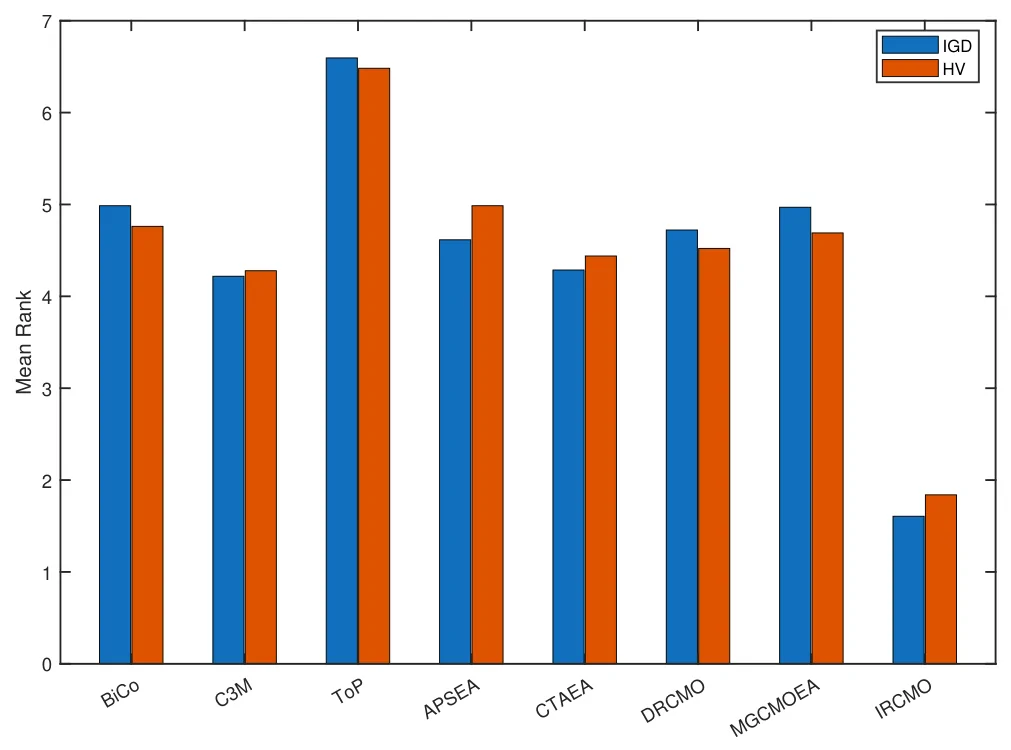
Average IGD and HV ranks of the eight algorithms over 59 problems.

Figure 4 reports the average ranks over 59 problems. IRCMO achieves the lowest average rank for both IGD and HV, indicating strong aggregate performance across different test suites. The pairwise Wilcoxon signed-rank results in Table 9 and Table 10 show that all *p*-values are below 0.05, with $R^{+}$ generally much larger than $R^{-}$. Although several comparison algorithms perform better on individual CF, DAS-CMOP, and LIR-CMOP instances, the overall assessment is based on aggregate performance rather than dominance on every problem. C3M, for example, has a relatively larger $R^{-}$ and remains competitive on some instances. Overall, the ranking and statistical-test results indicate a significant overall advantage of IRCMO while confirming that some competing methods are more effective on particular problem structures.

### 4.8. Ablation and Parameter Sensitivity Analysis

To evaluate the contributions of the core mechanisms of IRCMO and their internal components, seven ablation variants are constructed as listed in Table 11. IRCMO-A, IRCMO-B, and IRCMO-C examine the effects of ITNES, RCHRA, and their joint use. IRCMO-D evaluates the adaptive immune-tolerance mechanism by fixing the tolerance threshold at zero. IRCMO-E removes cross-population ecological complementarity from RCHRA. IRCMO-F replaces the squared excess-violation response in ITNES with a linear response, while IRCMO-G replaces the joint objective–decision niche distance with objective-space distance only. Except for the targeted modification, all variants use the same initialization, genetic operators, main-population environmental selection, function-evaluation budget, and parameter settings as the complete IRCMO.

Table 12 shows that the complete IRCMO achieves the lowest mean ranks of 1.1785 for IGD and 1.1071 for HV. Removing ITNES or RCHRA causes a clear performance loss, while removing both mechanisms further weakens the overall search capability. IRCMO-D obtains the largest mean ranks for both metrics, showing that fixing the tolerance threshold at zero substantially reduces the ability of the auxiliary population to preserve informative mildly infeasible candidates. IRCMO-E also performs noticeably worse than the complete algorithm, which confirms that reproductive success alone is insufficient to characterize the effective contribution of a population without considering cross-population complementarity. IRCMO-F shows a marked deterioration after replacing the squared excess-violation response with a linear response. This indicates that the nonlinear suppression of excess violation is important for maintaining a gradual transition between exploratory search and feasibility pressure. IRCMO-G also performs worse after removing the decision-space component from niche estimation, confirming that objective-space diversity alone cannot fully preserve structurally differentiated search directions. These results demonstrate that adaptive tolerance, nonlinear excess-violation regulation, joint objective–decision niche exclusion, and complementarity-aware resource allocation all contribute to the performance of IRCMO.

The sensitivity of the minimum resource protection setting in RCHRA is further examined in Table 13. The coefficient of *T* in $T_{\min}$ is varied among 0.05, 0.15, 0.25, 0.35, and 0.45, with 0.25 corresponding to the default setting used by IRCMO. The upper tested value is limited to 0.45 because the coefficient determines the minimum resource share reserved for each population. When it reaches 0.5, the lower and upper allocation bounds coincide, forcing the two populations to receive equal offspring budgets and eliminating the adaptive allocation capability of RCHRA. Values above 0.5 are therefore inconsistent with the bounded allocation rule. The default setting achieves the lowest mean rank for both IGD and HV. Smaller values weaken the protection of the population receiving fewer resources and allow the allocation to become more concentrated, whereas larger values restrict the ability of RCHRA to shift evaluations toward the population making the stronger current contribution. The results indicate that an intermediate protection level provides a better balance between adaptive resource redistribution and continued participation of both populations. The setting $T_{\min}=\max\left\{1,0.25T\right\}$ is therefore retained as the default configuration of IRCMO.

## 5. Conclusions

This paper proposes IRCMO to improve the use of infeasible search information and the allocation of evaluation resources in dual-population constrained multiobjective optimization. ITNES regulates auxiliary candidates through adaptive tolerance and joint-space niche selection, while RCHRA adjusts offspring budgets according to reproductive success and cross-population complementarity. Together, these mechanisms coordinate auxiliary exploration with adaptive search-resource allocation. The experimental results provide clear evidence of the effectiveness of IRCMO. It achieves the lowest overall average ranks for both IGD and HV, and the pairwise Wilcoxon tests against all seven comparison algorithms are significant at the 0.05 level. Its advantage is particularly consistent on the MW suite, where it obtains the best mean IGD and HV on all 14 problems. The results on CF, DAS-CMOP, and LIR-CMOP further show that IRCMO remains competitive across different constraint structures, although several specialized algorithms retain advantages on particular problems. These findings suggest that coordinated auxiliary exploration and adaptive resource allocation can improve robustness across heterogeneous constrained search landscapes.

IRCMO also has clear limitations. It fails to obtain feasible solutions reliably on RWMOP10, indicating limited feasibility recovery under extremely difficult feasible structures. The joint-distance computation and greedy truncation also result in relatively high worst-case computational cost. In addition, the present experiments do not establish scalability to large-scale CMOPs with very high-dimensional decision spaces. Future work will investigate stronger feasibility-recovery mechanisms, lighter niche and complementarity estimation, and scalable variants for larger decision spaces.

## Figures and Tables

**Table 1:** Comparison of representative constrained multiobjective evolutionary algorithms.

Method	Constraint Handling	Resource Allocation	Time Complexity	Reported Limitations
**Category I. Gradual-Evolution Methods**				
MOEA/D-DAE				
(N=100/105)	Improved ϵ handling with detect-and-escape regulation.	Utility-based subproblem activation.	Not explicitly reported	DAE is activated only once.
DSPCMDE				
(N=100)	Dynamic preference between Pareto dominance and CDP.	Fixed population and offspring sizes.	$O(MN^{2})$	Requires a predefined maximum generation number.
MOEA/D-2WA				
(*N* varies)	Constraint violation as an additional objective with a dynamic boundary.	Progressive reduction of infeasible weights.	Not explicitly reported	Sensitive to predefined weight distributions.
CMOEA-SDE				
(N=100)	Strict constrained dominance with constrained density estimation.	Fixed allocation.	$O(N^{2})$	May be affected by noncommensurate constraint violations.
ACR				
(N=100)	Adaptive *K*-nearest-neighbor regulation of constraint violation.	Dynamic population/archive mating ratio.	$O(N^{3})$	Scalability to many-objective and large-scale CMOPs is unverified.
**Category II. Multipopulation Cooperative Methods**				
BiCo				
(N=100)	CDP for the main population and angle-based selection of infeasible solutions.	Restricted mating without explicit resource reallocation.	Not explicitly reported	Less effective when the constrained and unconstrained PFs coincide.
MTCMO				
(N=100)	Dynamic auxiliary task with improved ϵ handling.	Fixed equal offspring allocation with knowledge transfer.	$O(MN^{2})$	May fail when all initial solutions are feasible.
DPACMO				
(N=100/105)	CDP with adaptive ϵ relaxation for bilateral CPF approximation.	Fixed equal offspring allocation with population migration.	Not explicitly reported	Scalability to large-scale and many-objective CMOPs is unverified.
DPCPRA				
(N=100)	CDP with progressive constraint processing in the auxiliary population.	Dynamic offspring allocation based on search feedback.	$O(MN^{2})$	Auxiliary constraint handling is less effective for large feasible regions.
MCCMO				
(N=100)	Separate searches with all constraints, no constraints, and subsets of constraints.	Population activation, dormancy, and constraint group merging.	$O(C^{2}MN^{2})$	Type II and Type III constraint relationships remain difficult to identify.
**Category III. Multistage Methods**				
PPS				
(N=300)	Constraint-ignoring push stage followed by improved ϵ handling.	Fixed population with search-state-based stage switching.	Not explicitly reported	Unsuitable when the unconstrained PF collapses to one point.
C-TSEA				
(N=91)	Constraint-ignoring first stage followed by archive-assisted CDP search.	Fixed population with a predefined stage ratio.	$O(N^{3})$	Limited exploration of corner and sparse feasible regions.
KTS				
(N=100)	Adaptive switching between unconstrained and constrained surrogate-assisted search.	Fixed infill budget with adaptive search-mode switching.	Not explicitly reported	Kriging limits the tested decision dimension to 10.
C3M				
(N=100/105)	Progressive constraint handling according to constraint priority.	Skips less important constraint evaluations across stages.	$O(CMN^{2})$	Constraint-priority estimation can be incorrect.
CMOES				
(N=100)	Constraint-ignoring first stage followed by parameter-free FNDS handling.	Fixed population with a two-stage search schedule.	$O(MN^{2})$	Not explicitly reported.
**Category IV. Hybrid Methods**				
URCMO				
(N=100)	CDP-based main search with learning of the relationship between CPF and UPF.	Fixed population budgets with relation-guided auxiliary strategies.	Not explicitly reported	Problem-type classification may be inaccurate for highly complex CMOPs.
CMOQLMT				
(N=100)	SPEA2-CDP with multiple auxiliary constraint-handling strategies.	Q-learning-based adaptive auxiliary-task selection.	Not explicitly reported	Performance depends on the embedded auxiliary strategies.
SC-EMT				
(N=100)	Main-task constraints with auxiliary constraint-subset handling.	Fixed per-task offspring allocation with stage-based scheduling.	$O(mMN^{2})$	Constraint grouping ignores interconstraint correlations.
BPRRA				
(N=100)	Constraint-ignoring and CDP boundary search followed by ICMA and CDP.	Offspring-contribution-based dynamic resource allocation.	$O(N^{3})$	Resource allocation requires further generalization.
DPTPEA				
(N=100/105)	CDP main search with unconstrained and ϵ-constrained tractive search.	Feasible-rate-based dynamic cooperation with fixed total offspring.	Not explicitly reported	Scalability to large-scale CMOPs is unverified.

**Table 2:** Main characteristics of the benchmark test suites used in the experiments.

Test Suite	Decision Variables	Objectives	Variable Type/Problem Nature
CF	D=10	2: CF1–CF7 3: CF8–CF10	Continuous real-valued Nonlinear
DAS-CMOP	D=30	2: DAS-CMOP1–6 3: DAS-CMOP7–9	Continuous real-valued Nonlinear
LIR-CMOP	D=30	2: LIR-CMOP1–12 3: LIR-CMOP13–14	Continuous real-valued Nonlinear
MW	D=15	2: MW1–3, MW5–7, MW9–13 3: MW4, MW8, MW14	Continuous real-valued Nonlinear

**Table 3:** IGD results of the compared algorithms on the 47 benchmark problems.

	BiCo	C3M	ToP	APSEA	C-TAEA	DRCMO	MGCMOEA	IRCMO
CF1	3.82e−02 (2.35e−03)+	**4.96e−03** **(8.54e−04)−**	6.83e−03 (1.03e−03)−	3.40e−02 (3.02e−03)+	6.63e−02 (1.50e−03)+	1.18e−02 (2.28e−03)=	1.10e−02 (1.56e−03)−	1.22e−02 (5.29e−04)
CF2	1.35e−01 (2.67e−02)+	6.38e−02 (3.99e−03)−	9.52e−02 (7.53e−02)=	1.37e−01 (2.22e−02)+	1.21e−01 (2.47e−02)+	4.19e−02 (1.16e−02)−	**4.14e−02** **(1.46e−02)−**	6.81e−02 (6.05e−03)
CF3	4.02e−01 (1.30e−01)+	4.07e−01 (4.65e−02)+	6.03e−01 (2.28e−01)+	4.63e−01 (1.57e−01)+	4.28e−01 (1.46e−01)+	5.08e−01 (2.60e−01)+	5.22e−01 (2.42e−01)+	**8.94e−02** **(2.06e−02)**
CF4	3.72e−01 (8.68e−02)+	3.60e−01 (1.11e−01)+	4.57e−01 (8.81e−02)+	4.68e−01 (9.80e−02)+	4.29e−01 (8.47e−02)+	3.73e−01 (1.84e−01)+	4.22e−01 (1.44e−01)+	**1.05e−01** **(5.45e−03)**
CF5	6.22e−01 (2.58e−02)+	3.82e+00 (6.11e−01)+	4.15e+00 (4.74e−01)+	5.98e−01 (4.02e−02)+	6.12e−01 (3.69e−02)+	4.29e−01 (1.28e−01)+	4.21e−01 (1.04e−01)+	**2.40e−01** **(5.69e−02)**
CF6	4.57e−01 (3.46e−02)+	2.22e−01 (3.59e−02)+	2.63e−01 (2.51e−02)+	4.16e−01 (3.28e−02)+	4.26e−01 (2.71e−02)+	2.55e−01 (8.36e−02)+	2.64e−01 (4.52e−02)+	**1.19e−01** **(2.79e−02)**
CF7	5.60e−01 (8.32e−02)+	6.30e+00 (6.89e−01)+	5.97e+00 (1.02e+00)+	6.20e−01 (9.39e−02)+	6.38e−01 (9.79e−02)+	5.13e−01 (1.76e−01)+	5.41e−01 (1.33e−01)+	**1.91e−01** **(2.62e−02)**
CF8	NaN (0.00%)+	7.60e−01 (9.35e−02)+	NaN (0.00%)+	5.39e−01 (1.87e−02)+	6.53e−01 (5.06e−02)+	NaN (44.40%)+	NaN (30.00%)+	**3.43e−01** **(5.11e−02)**
CF9	9.36e−01 (8.77e−03)+	3.06e−01 (3.47e−02)+	6.10e−01 (4.95e−02)+	2.01e−01 (6.81e−03)+	2.64e−01 (2.06e−02)+	6.51e−01 (3.80e−01)+	6.99e−01 (3.47e−01)+	**1.43e−01** **(2.17e−02)**
CF10	NaN (0.00%)+	2.94e+00 (1.01e+00)+	NaN (0.00%)+	4.48e−01 (7.79e−02)+	1.15e+00 (5.94e−01)+	NaN (0.00%)+	NaN (0.00%)+	**3.36e−01** **(4.77e−02)**
+/−/=	10/0/0	8/2/0	8/1/1	10/0/0	10/0/0	8/1/1	8/2/0	
DAS- CMOP1	7.82e−01 (9.10e−03)+	**1.29e−02** **(7.49e−03)−**	8.45e−01 (1.09e−02)+	7.94e−01 (1.30e−02)+	2.18e−01 (2.52e−02)−	7.52e−01 (3.34e−02)+	6.91e−01 (1.69e−01)+	6.51e−01 (3.94e−02)
DAS- CMOP2	2.77e−01 (1.29e−02)+	**5.28e−03** **(1.42e−04)−**	8.15e−01 (1.21e−02)+	2.88e−01 (1.53e−02)+	1.51e−01 (1.47e−02)−	2.54e−01 (4.68e−02)+	2.46e−01 (3.14e−02)+	2.07e−01 (1.58e−02)
DAS- CMOP3	3.99e−01 (5.01e−02)+	2.65e−01 (8.08e−04)+	8.23e−01 (5.62e−03)+	3.54e−01 (2.47e−02)+	2.50e−01 (1.89e−02)=	4.12e−01 (4.81e−02)+	4.14e−01 (9.23e−02)+	**2.43e−01** **(1.45e−02)**
DAS- CMOP4	4.10e−01 (8.57e−02)+	4.76e−01 (3.16e−01)+	NaN (0.00%)+	8.52e−02 (1.08e−01)+	1.55e−02 (1.08e−03)+	2.64e−01 (1.25e−01)+	1.99e−01 (1.62e−01)+	**1.16e−03** **(1.07e−05)**
DAS- CMOP5	5.02e−01 (6.86e−02)+	3.28e−01 (3.07e−01)+	NaN (0.00%)+	3.68e−01 (8.18e−02)+	8.97e−03 (4.43e−04)+	3.72e−01 (2.30e−01)+	2.52e−01 (2.24e−01)+	**2.73e−03** **(5.00e−05)**
DAS- CMOP6	5.49e−01 (9.91e−02)+	6.13e−01 (2.65e−01)+	NaN (0.00%)+	5.66e−01 (7.74e−02)+	3.71e−02 (7.21e−03)+	3.94e−01 (2.17e−01)+	4.05e−01 (2.47e−01)+	**1.64e−02** **(3.42e−03)**
DAS- CMOP7	3.94e−02 (2.18e−02)+	2.80e−01 (2.57e−01)+	NaN (0.00%)+	3.19e−02 (4.62e−04)+	4.87e−02 (2.45e−02)+	3.73e−02 (6.00e−03)+	3.76e−02 (9.80e−03)+	**3.05e−02** **(6.81e−04)**
DAS- CMOP8	4.48e−02 (4.74e−03)+	4.02e−01 (2.44e−01)+	NaN (0.00%)+	4.09e−02 (5.72e−04)+	6.53e−02 (1.53e−02)+	4.54e−02 (5.65e−03)+	4.76e−02 (1.42e−02)+	**3.87e−02** **(9.41e−04)**
DAS- CMOP9	4.45e−01 (2.25e−02)+	**4.62e−02** **(1.77e−03)−**	8.86e−01 (1.29e−02)+	4.74e−01 (3.70e−02)+	3.56e−01 (2.10e−02)+	3.82e−01 (1.71e−01)+	4.21e−01 (7.32e−02)+	2.46e−01 (2.78e−02)
+/−/=	9/0/0	6/3/0	9/0/0	9/0/0	6/2/1	9/0/0	9/0/0	
LIR- CMOP1	2.44e−01 (7.66e−03)+	3.00e−01 (3.35e−02)+	3.46e−01 (1.10e−02)+	3.44e−01 (1.29e−02)+	2.12e−01 (7.92e−02)=	3.20e−01 (3.13e−02)+	3.07e−01 (3.06e−02)+	**1.97e−01** **(1.40e−02)**
LIR- CMOP2	2.10e−01 (8.40e−03)+	2.71e−01 (3.04e−02)+	3.02e−01 (1.01e−02)+	2.90e−01 (1.48e−02)+	1.75e−01 (3.61e−02)+	2.56e−01 (3.52e−02)+	2.51e−01 (2.89e−02)+	**1.54e−01** **(1.22e−02)**
LIR- CMOP3	2.62e−01 (1.67e−02)+	3.94e−01 (8.98e−02)+	3.50e−01 (4.97e−03)+	3.40e−01 (8.10e−03)+	2.87e−01 (7.45e−02)+	3.12e−01 (3.50e−02)+	3.12e−01 (4.59e−02)+	**1.67e−01** **(1.92e−02)**
LIR- CMOP4	2.53e−01 (5.67e−03)+	3.49e−01 (5.62e−02)+	3.35e−01 (1.11e−02)+	3.16e−01 (8.84e−03)+	2.21e−01 (5.27e−02)+	3.02e−01 (3.54e−02)+	2.96e−01 (3.15e−02)+	**1.88e−01** **(2.21e−02)**
LIR- CMOP5	1.23e+00 (1.87e−03)+	5.64e−01 (4.85e−01)=	1.23e+00 (4.17e−03)+	1.22e+00 (2.21e−03)+	1.29e+00 (2.45e−01)+	4.04e−01 (8.28e−02)+	9.91e−01 (3.99e−01)+	**2.82e−01** **(1.14e−02)**
LIR- CMOP6	1.35e+00 (7.79e−05)+	3.71e−01 (3.59e−01)=	1.35e+00 (1.08e−03)+	1.35e+00 (1.50e−04)+	1.35e+00 (6.41e−04)+	4.83e−01 (1.34e−01)+	1.26e+00 (2.68e−01)+	**3.35e−01** **(2.95e−02)**
LIR- CMOP7	1.68e+00 (3.05e−04)+	8.68e−02 (1.75e−01)−	1.68e+00 (1.65e−03)+	1.65e−01 (1.65e−02)+	1.10e+00 (5.85e−01)+	1.89e−01 (3.66e−02)+	1.07e+00 (7.65e−01)+	**6.19e−02** **(9.52e−03)**
LIR- CMOP8	1.68e+00 (1.73e−04)+	**4.06e−02** **(4.36e−02)−**	1.69e+00 (4.70e−03)+	2.59e−01 (3.27e−02)+	1.69e+00 (9.52e−04)+	3.00e−01 (7.83e−02)+	1.16e+00 (6.98e−01)+	8.24e−02 (1.19e−02)
LIR- CMOP9	1.06e+00 (1.26e−02)+	4.78e−01 (4.07e−02)+	7.47e−01 (2.82e−02)+	9.99e−01 (2.25e−02)+	6.31e−01 (3.99e−02)+	5.92e−01 (1.05e−01)+	6.03e−01 (1.08e−01)+	**4.09e−01** **(4.13e−02)**
LIR- CMOP10	1.03e+00 (2.01e−02)+	**2.74e−01** **(8.46e−02)−**	5.87e−01 (1.35e−01)+	9.54e−01 (4.65e−02)+	4.35e−01 (2.06e−02)+	5.31e−01 (2.14e−01)+	5.89e−01 (2.13e−01)+	3.40e−01 (7.22e−02)
LIR- CMOP11	9.20e−01 (4.78e−02)+	2.00e−01 (8.67e−02)+	6.17e−01 (5.49e−02)+	7.54e−01 (2.29e−03)+	3.15e−01 (1.41e−02)+	5.29e−01 (1.40e−01)+	5.68e−01 (1.69e−01)+	**1.08e−01** **(3.00e−02)**
LIR- CMOP12	9.52e−01 (7.03e−02)+	1.85e−01 (3.87e−02)+	3.90e−01 (4.71e−02)+	6.98e−01 (1.24e−01)+	5.30e−01 (7.99e−02)+	3.49e−01 (8.22e−02)+	4.25e−01 (6.72e−02)+	**1.09e−01** **(1.66e−02)**
LIR- CMOP13	1.32e+00 (1.09e−03)+	2.52e−01 (2.39e−01)−	1.35e+00 (3.54e−03)+	1.32e+00 (1.39e−03)−	1.13e−01 (2.88e−03)−	**1.04e−01** **(1.53e−03)−**	7.03e−01 (6.19e−01)−	1.32e+00 (2.03e−03)
LIR- CMOP14	1.28e+00 (1.55e−03)+	2.44e−01 (2.73e−01)−	1.31e+00 (4.26e−03)+	1.27e+00 (1.15e−03)−	**1.13e−01** **(8.38e−04)−**	1.30e−01 (9.74e−02)−	8.79e−01 (5.61e−01)−	1.27e+00 (1.24e−03)
+/−/=	14/0/0	7/5/2	14/0/0	12/2/0	11/2/1	12/2/0	12/2/0	
MW1	8.10e−02 (1.39e−01)+	NaN (0.00%)+	NaN (0.00%)+	2.09e−02 (5.14e−02)+	5.41e−03 (9.32e−03)+	3.97e−01 (2.61e−01)+	NaN (91.57%)+	**1.76e−03** **(5.74e−05)**
MW2	4.21e−02 (7.62e−03)+	3.57e−01 (4.91e−02)+	4.02e−01 (1.64e−01)+	7.24e−02 (3.02e−02)+	5.65e−02 (2.62e−02)+	3.61e−01 (3.48e−02)+	4.11e−01 (1.59e−01)+	**1.86e−02** **(2.01e−03)**
MW3	8.97e−03 (6.19e−04)+	1.38e−02 (2.35e−03)+	NaN (0.00%)+	8.01e−03 (6.77e−04)+	7.37e−03 (4.91e−04)+	1.65e−02 (3.33e−03)+	9.19e−01 (6.09e−08)+	**5.66e−03** **(2.14e−04)**
MW4	8.56e−02 (1.16e−01)+	NaN (6.67%)+	NaN (0.00%)+	5.00e−02 (2.56e−02)+	5.00e−02 (6.14e−03)+	1.85e−01 (2.12e−01)+	2.94e−01 (2.77e−01)+	**4.13e−02** **(2.88e−04)**
MW5	7.42e−01 (2.44e−02)+	NaN (3.33%)+	NaN (0.00%)+	1.69e−01 (1.58e−01)+	3.76e−02 (3.01e−02)+	7.73e−01 (6.08e−02)+	7.55e−01 (2.84e−03)+	**8.04e−04** **(1.80e−04)**
MW6	4.72e−02 (2.57e−02)+	1.20e+00 (7.38e−02)+	1.37e+00 (4.67e−02)+	3.50e−01 (1.92e−01)+	6.68e−02 (8.41e−02)+	1.18e+00 (1.07e−01)+	1.25e+00 (9.17e−02)+	**1.85e−02** **(3.39e−03)**
MW7	8.33e−03 (4.88e−04)+	9.72e−03 (6.22e−04)+	6.74e−01 (9.23e−02)+	7.27e−03 (6.10e−04)+	8.23e−03 (4.49e−04)+	1.11e−02 (6.71e−04)+	1.03e−02 (6.18e−04)+	**4.96e−03** **(3.22e−04)**
MW8	5.55e−02 (4.28e−03)+	5.02e−01 (3.80e−01)+	1.06e+00 (5.55e−02)+	8.61e−02 (2.47e−02)+	8.20e−02 (2.03e−02)+	4.93e−01 (3.81e−01)+	6.21e−01 (2.87e−01)+	**4.84e−02** **(1.13e−03)**
MW9	7.15e−01 (1.08e−02)+	NaN (13.37%)+	NaN (0.00%)+	7.18e−01 (2.64e−02)+	7.12e−01 (4.82e−03)+	7.29e−01 (2.96e−02)+	7.16e−01 (1.81e−02)+	**4.45e−03** **(1.41e−04)**
MW10	1.60e−01 (3.96e−02)+	NaN (26.67%)+	NaN (4.70%)+	2.74e−01 (1.42e−01)+	1.20e−01 (1.03e−01)+	6.28e−01 (3.61e−02)+	6.52e−01 (3.67e−02)+	**2.72e−02** **(7.14e−03)**
MW11	7.99e−02 (1.91e−01)+	7.59e−03 (2.61e−04)+	NaN (24.33%)+	7.17e−03 (5.36e−03)+	1.54e−02 (9.32e−04)+	7.89e−03 (4.82e−04)+	7.51e−03 (3.43e−04)+	**5.88e−03** **(1.75e−04)**
MW12	7.74e−01 (2.63e−03)+	NaN (6.67%)+	NaN (0.00%)+	7.75e−01 (4.24e−03)+	7.82e−01 (4.55e−03)+	8.08e−01 (7.31e−02)+	7.92e−01 (1.44e−01)+	**4.81e−03** **(7.86e−05)**
MW13	1.65e−01 (2.51e−01)+	1.69e+00 (1.75e−01)+	1.67e+00 (9.94e−02)+	2.15e−01 (7.29e−02)+	1.28e−01 (2.45e−02)+	1.74e+00 (1.38e−01)+	1.60e+00 (2.69e−01)+	**6.93e−02** **(9.18e−03)**
MW14	4.22e−01 (6.60e−02)+	7.32e−01 (3.95e−02)+	2.55e+00 (3.42e−02)+	3.52e−01 (1.06e−01)+	3.67e−01 (7.54e−02)+	4.46e−01 (2.62e−02)+	4.65e−01 (2.58e−02)+	**1.24e−01** **(1.43e−02)**
+/−/=	14/0/0	14/0/0	14/0/0	14/0/0	14/0/0	14/0/0	14/0/0	

**Table 4:** HV results of the compared algorithms on the 47 benchmark problems.

	BiCo	C3M	ToP	APSEA	C-TAEA	DRCMO	MGCMOEA	IRCMO
CF1	5.18e−01 (2.84e−03)+	**5.60e−01** **(1.04e−03)−**	5.58e−01 (1.37e−03)−	5.23e−01 (3.62e−03)+	4.90e−01 (1.45e−03)+	5.52e−01 (2.78e−03)−	5.53e−01 (1.88e−03)−	5.50e−01 (7.20e−04)
CF2	5.13e−01 (1.83e−02)+	5.90e−01 (7.52e−03)+	5.76e−01 (2.61e−02)+	5.18e−01 (1.97e−02)+	5.21e−01 (2.20e−02)+	6.16e−01 (2.06e−02)−	**6.24e−01** **(1.42e−02)−**	5.98e−01 (1.58e−02)
CF3	1.50e−01 (3.77e−02)+	4.48e−02 (1.32e−02)+	7.26e−02 (1.36e−02)+	1.25e−01 (4.00e−02)+	1.27e−01 (3.69e−02)+	1.21e−01 (6.71e−02)+	1.17e−01 (6.54e−02)+	**2.73e−01** **(1.20e−02)**
CF4	2.37e−01 (5.84e−02)+	1.98e−01 (6.70e−02)+	1.54e−01 (4.39e−02)+	1.90e−01 (5.17e−02)+	2.13e−01 (4.52e−02)+	2.26e−01 (1.23e−01)+	1.91e−01 (8.54e−02)+	**4.12e−01** **(3.11e−03)**
CF5	1.13e−01 (2.58e−02)+	0.00e+00 (0.00e+00)+	0.00e+00 (0.00e+00)+	1.31e−01 (2.48e−02)+	1.16e−01 (1.93e−02)+	1.58e−01 (8.76e−02)+	1.68e−01 (7.45e−02)+	**3.06e−01** **(2.22e−02)**
CF6	3.62e−01 (2.21e−02)+	4.84e−01 (1.45e−02)+	4.55e−01 (1.20e−02)+	3.88e−01 (1.80e−02)+	3.81e−01 (1.48e−02)+	5.00e−01 (5.20e−02)+	4.91e−01 (3.19e−02)+	**5.84e−01** **(8.02e−03)**
CF7	2.27e−01 (9.27e−02)+	0.00e+00 (0.00e+00)+	0.00e+00 (0.00e+00)+	1.79e−01 (7.38e−02)+	1.95e−01 (7.90e−02)+	2.20e−01 (1.28e−01)+	1.82e−01 (1.09e−01)+	**4.89e−01** **(4.24e−02)**
CF8	NaN (0.00%)+	1.33e−02 (6.11e−03)+	NaN (0.00%)+	1.19e−01 (9.65e−03)+	9.90e−02 (1.61e−02)+	NaN (44.40%)+	NaN (30.00%)+	**1.72e−01** **(2.15e−02)**
CF9	9.09e−02 (1.68e−06)+	1.23e−01 (2.69e−02)+	1.60e−02 (1.49e−02)+	2.58e−01 (8.51e−03)+	1.74e−01 (1.65e−02)+	1.67e−01 (1.04e−01)+	1.46e−01 (8.89e−02)+	**3.27e−01** **(2.66e−02)**
CF10	NaN (0.00%)+	0.00e+00 (0.00e+00)+	NaN (0.00%)+	8.62e−02 (3.66e−03)+	1.22e−02 (1.77e−02)+	NaN (0.00%)+	NaN (0.00%)+	**1.36e−01** **(2.82e−02)**
+/−/=	10/0/0	9/1/0	9/1/0	10/0/0	10/0/0	8/2/0	8/2/0	
DAS- CMOP1	0.00e+00 (0.00e+00)+	**2.09e−01** **(1.03e−03)−**	0.00e+00 (0.00e+00)+	0.00e+00 (0.00e+00)+	1.58e−01 (1.39e−02)−	3.53e−03 (5.49e−03)+	1.83e−02 (4.67e−02)+	4.44e−02 (1.92e−02)
DAS- CMOP2	2.47e−01 (2.37e−03)+	**3.54e−01** **(1.24e−04)−**	2.05e−02 (2.32e−03)+	2.47e−01 (2.07e−03)+	2.89e−01 (3.91e−03)−	2.54e−01 (1.38e−02)+	2.55e−01 (7.48e−03)+	2.65e−01 (5.12e−03)
DAS- CMOP3	2.08e−01 (1.96e−04)+	**2.53e−01** **(2.43e−04)−**	1.49e−03 (1.90e−03)+	2.08e−01 (2.69e−04)+	2.28e−01 (9.23e−03)+	2.05e−01 (1.74e−02)+	2.01e−01 (3.80e−02)+	**2.41e−01** **(8.08e−03)**
DAS- CMOP4	5.52e−02 (1.39e−02)+	6.17e−02 (6.88e−02)+	NaN (0.00%)+	1.69e−01 (3.59e−02)+	1.88e−01 (1.06e−03)+	9.70e−02 (5.13e−02)+	1.22e−01 (6.46e−02)+	**2.04e−01** **(5.59e−05)**
DAS- CMOP5	7.42e−02 (1.76e−02)+	1.90e−01 (1.45e−01)+	NaN (0.00%)+	1.28e−01 (3.77e−02)+	3.47e−01 (3.54e−04)+	1.48e−01 (1.23e−01)+	2.06e−01 (1.24e−01)+	**3.52e−01** **(9.86e−05)**
DAS- CMOP6	5.38e−02 (2.14e−02)+	6.68e−02 (7.41e−02)+	NaN (0.00%)+	4.73e−02 (1.48e−02)+	3.03e−01 (4.01e−03)+	1.20e−01 (1.08e−01)+	1.22e−01 (1.13e−01)+	**3.12e−01** **(4.03e−04)**
DAS- CMOP7	2.82e−01 (1.25e−02)+	1.82e−01 (6.77e−02)+	NaN (0.00%)+	2.88e−01 (2.22e−04)+	2.84e−01 (7.60e−03)+	2.84e−01 (2.46e−03)+	2.83e−01 (4.22e−03)+	**2.88e−01** **(3.33e−04)**
DAS- CMOP8	2.04e−01 (3.38e−03)+	8.15e−02 (5.21e−02)+	NaN (0.00%)+	2.07e−01 (2.25e−04)+	2.01e−01 (3.41e−03)+	2.06e−01 (1.21e−03)+	2.04e−01 (4.07e−03)+	**2.07e−01** **(5.94e−04)**
DAS- CMOP9	1.10e−01 (5.83e−03)+	**2.03e−01** **(7.42e−04)−**	4.21e−02 (3.93e−04)+	1.10e−01 (5.92e−03)+	1.26e−01 (3.15e−03)+	1.31e−01 (3.58e−02)+	1.21e−01 (1.32e−02)+	1.48e−01 (5.37e−03)
+/−/=	9/0/0	5/4/0	9/0/0	9/0/0	7/2/0	9/0/0	9/0/0	
LIR- CMOP1	1.27e−01 (3.25e−03)+	1.12e−01 (6.97e−03)+	9.37e−02 (5.21e−03)+	9.79e−02 (5.95e−03)+	1.42e−01 (2.07e−02)=	1.07e−01 (1.39e−02)+	1.10e−01 (1.12e−02)+	**1.44e−01** **(3.77e−03)**
LIR- CMOP2	2.46e−01 (4.95e−03)+	2.04e−01 (1.48e−02)+	1.99e−01 (8.56e−03)+	2.12e−01 (7.50e−03)+	**2.84e−01** **(2.16e−02)−**	2.24e−01 (2.07e−02)+	2.29e−01 (1.45e−02)+	**2.81e−01** **(6.98e−03)**
LIR- CMOP3	1.10e−01 (3.36e−03)+	9.32e−02 (1.12e−02)+	8.53e−02 (4.76e−03)+	9.10e−02 (4.55e−03)+	1.14e−01 (2.02e−02)+	9.57e−02 (1.03e−02)+	9.74e−02 (1.56e−02)+	**1.45e−01** **(4.64e−03)**
LIR- CMOP4	2.08e−01 (5.97e−03)+	1.70e−01 (2.58e−02)+	1.63e−01 (7.06e−03)+	1.78e−01 (7.03e−03)+	2.27e−01 (2.67e−02)=	1.84e−01 (1.80e−02)+	1.86e−01 (1.66e−02)+	**2.37e−01** **(8.78e−03)**
LIR- CMOP5	0.00e+00 (0.00e+00)+	1.37e−01 (1.11e−01)=	0.00e+00 (0.00e+00)+	0.00e+00 (0.00e+00)+	0.00e+00 (0.00e+00)+	1.13e−01 (2.95e−02)+	3.67e−02 (6.21e−02)+	**1.68e−01** **(9.80e−03)**
LIR- CMOP6	0.00e+00 (0.00e+00)+	**9.95e−02** **(5.16e−02)=**	0.00e+00 (0.00e+00)+	0.00e+00 (0.00e+00)+	0.00e+00 (0.00e+00)+	8.57e−02 (1.29e−02)+	8.53e−03 (2.65e−02)+	**9.93e−02** **(8.58e−03)**
LIR- CMOP7	0.00e+00 (0.00e+00)+	**2.69e−01** **(4.27e−02)−**	0.00e+00 (0.00e+00)+	2.35e−01 (4.62e−03)+	7.89e−02 (8.09e−02)+	2.29e−01 (9.83e−03)+	9.59e−02 (1.20e−01)+	**2.66e−01** **(3.41e−03)**
LIR- CMOP8	0.00e+00 (0.00e+00)+	**2.81e−01** **(1.57e−02)−**	0.00e+00 (0.00e+00)+	2.20e−01 (2.95e−03)+	0.00e+00 (0.00e+00)+	2.16e−01 (7.63e−03)+	8.04e−02 (1.08e−01)+	2.60e−01 (3.56e−03)
LIR- CMOP9	8.63e−02 (2.85e−03)+	3.68e−01 (1.49e−02)+	2.06e−01 (1.30e−02)+	1.05e−01 (2.07e−02)+	2.86e−01 (1.38e−02)+	3.02e−01 (6.08e−02)+	2.97e−01 (6.89e−02)+	**3.95e−01** **(3.17e−02)**
LIR- CMOP10	5.26e−02 (1.63e−04)+	**5.70e−01** **(5.02e−02)−**	3.05e−01 (1.37e−01)+	5.61e−02 (6.93e−03)+	4.66e−01 (1.88e−02)+	3.35e−01 (1.82e−01)+	2.92e−01 (1.79e−01)+	5.08e−01 (4.67e−02)
LIR- CMOP11	1.72e−01 (1.09e−02)+	5.65e−01 (5.77e−02)+	2.65e−01 (2.06e−02)+	2.26e−01 (2.95e−03)+	5.56e−01 (7.21e−03)+	3.24e−01 (1.11e−01)+	3.26e−01 (1.24e−01)+	**6.51e−01** **(8.59e−03)**
LIR- CMOP12	1.82e−01 (1.52e−02)+	5.23e−01 (1.94e−02)+	4.22e−01 (1.70e−02)+	3.10e−01 (7.38e−02)+	4.12e−01 (2.14e−02)+	4.28e−01 (5.42e−02)+	3.70e−01 (4.19e−02)+	**5.73e−01** **(9.86e−03)**
LIR- CMOP13	0.00e+00 (0.00e+00)+	4.06e−01 (1.02e−01)−	0.00e+00 (0.00e+00)+	0.00e+00 (0.00e+00)+	**5.44e−01** **(1.79e−03)−**	**5.34e−01** **(2.24e−03)−**	2.77e−01 (2.81e−01)=	2.43e−04 (1.41e−04)
LIR- CMOP14	1.45e−04 (1.35e−04)+	4.41e−01 (1.36e−01)−	0.00e+00 (0.00e+00)+	2.11e−04 (2.14e−04)+	**5.45e−01** **(7.88e−04)−**	5.28e−01 (6.37e−02)−	1.84e−01 (2.64e−01)=	7.27e−04 (3.30e−04)
+/−/=	14/0/0	7/5/2	14/0/0	14/0/0	9/3/2	12/2/0	12/0/2	
MW1	4.20e−01 (1.07e−01)+	NaN (0.00%)+	NaN (0.00%)+	4.67e−01 (5.41e−02)+	4.83e−01 (1.16e−02)+	1.78e−01 (1.70e−01)+	NaN (91.57%)+	**4.89e−01** **(2.19e−04)**
MW2	5.22e−01 (1.06e−02)+	1.90e−01 (2.92e−02)+	1.85e−01 (1.10e−01)+	4.81e−01 (3.80e−02)+	5.02e−01 (3.28e−02)+	1.86e−01 (2.06e−02)+	1.68e−01 (4.93e−02)+	**5.56e−01** **(3.11e−03)**
MW3	5.37e−01 (9.02e−04)+	5.27e−01 (4.54e−03)+	NaN (0.00%)+	5.39e−01 (1.08e−03)+	5.40e−01 (7.28e−04)+	5.25e−01 (6.20e−03)+	0.00e+00 (0.00e+00)+	**5.43e−01** **(3.22e−04)**
MW4	7.82e−01 (1.47e−01)+	NaN (6.67%)+	NaN (0.00%)+	8.27e−01 (4.30e−02)=	8.34e−01 (6.84e−03)+	6.66e−01 (2.17e−01)+	5.64e−01 (2.39e−01)+	**8.40e−01** **(3.96e−04)**
MW5	8.78e−02 (1.66e−02)+	NaN (3.33%)+	NaN (0.00%)+	2.52e−01 (5.61e−02)+	2.98e−01 (2.42e−02)+	7.38e−02 (3.12e−02)+	8.94e−02 (4.67e−03)+	**3.24e−01** **(1.56e−04)**
MW6	2.70e−01 (2.07e−02)+	0.00e+00 (0.00e+00)+	0.00e+00 (0.00e+00)+	1.65e−01 (5.36e−02)+	2.61e−01 (3.34e−02)+	0.00e+00 (0.00e+00)+	0.00e+00 (0.00e+00)+	**3.05e−01** **(4.61e−03)**
MW7	4.05e−01 (7.87e−04)+	4.04e−01 (1.43e−03)+	4.83e−02 (5.21e−02)+	4.07e−01 (1.00e−03)+	4.06e−01 (1.04e−03)+	4.02e−01 (9.47e−04)+	4.03e−01 (8.13e−04)+	**4.11e−01** **(3.40e−04)**
MW8	5.06e−01 (1.03e−02)+	1.60e−01 (1.20e−01)+	2.01e−03 (6.25e−03)+	4.42e−01 (4.46e−02)+	4.48e−01 (4.07e−02)+	1.40e−01 (9.61e−02)+	9.01e−02 (7.04e−02)+	**5.27e−01** **(4.48e−03)**
MW9	0.00e+00 (0.00e+00)+	NaN (13.37%)+	NaN (0.00%)+	0.00e+00 (0.00e+00)+	0.00e+00 (0.00e+00)+	0.00e+00 (0.00e+00)+	0.00e+00 (0.00e+00)+	**4.00e−01** **(1.22e−03)**
MW10	3.39e−01 (2.18e−02)+	NaN (26.67%)+	NaN (4.70%)+	2.83e−01 (6.51e−02)+	3.64e−01 (5.13e−02)+	1.21e−01 (1.77e−02)+	1.08e−01 (1.62e−02)+	**4.25e−01** **(6.80e−03)**
MW11	4.26e−01 (5.14e−02)+	4.46e−01 (2.44e−04)+	NaN (24.33%)+	4.47e−01 (1.30e−03)+	4.42e−01 (5.08e−04)+	4.46e−01 (3.44e−04)+	4.47e−01 (2.66e−04)+	**4.48e−01** **(1.62e−04)**
MW12	0.00e+00 (0.00e+00)+	NaN (6.67%)+	NaN (0.00%)+	0.00e+00 (0.00e+00)+	0.00e+00 (0.00e+00)+	0.00e+00 (0.00e+00)+	4.48e−03 (7.50e−03)+	**6.05e−01** **(1.50e−04)**
MW13	4.10e−01 (4.38e−02)+	0.00e+00 (0.00e+00)+	9.83e−02 (2.54e−02)+	3.52e−01 (4.15e−02)+	4.11e−01 (1.85e−02)+	0.00e+00 (0.00e+00)+	7.46e−03 (1.88e−02)+	**4.48e−01** **(3.77e−03)**
MW14	3.44e−01 (2.98e−02)+	1.77e−01 (1.42e−02)+	1.33e−02 (7.85e−04)+	3.79e−01 (5.61e−02)+	3.75e−01 (3.46e−02)+	3.27e−01 (1.47e−02)+	3.15e−01 (1.45e−02)+	**4.54e−01** **(5.69e−03)**
+/−/=	14/0/0	14/0/0	14/0/0	13/0/1	14/0/0	14/0/0	14/0/0	

**Table 5:** Basic information for the 12 real-world constrained multiobjective optimization problems.

Problem	Name	M	D
RWMOP1	Vibrating Platform Design	2	5
RWMOP2	Welded Beam Design	2	4
RWMOP3	Speed Reducer Design	2	7
RWMOP4	Gear Train Design	2	4
RWMOP5	Four Bar Plane Truss	2	4
RWMOP6	Simply Supported I-beam Design	2	4
RWMOP7	Multiple Disk Clutch Brake Design	2	5
RWMOP8	Spring Design	2	3
RWMOP9	Crash Energy Management for High-speed Train	2	6
RWMOP10	Haverly’s Pooling Problem	2	9
RWMOP11	Process Synthesis Problem	2	2
RWMOP12	Process Synthesis and Design Problem	2	3

**Table 6:** IGD results of the compared algorithms on the 12 real-world problems.

	BiCo	C3M	ToP	APSEA	C-TAEA	DRCMO	MGCMOEA	IRCMO
RW- MOP1	9.15e+01 (3.69e−02)+	9.15e+01 (5.28e−03)+	9.14e+01 (1.20e−01)+	8.66e+01 (1.82e+01)+	1.48e+02 (4.84e+01)+	9.15e+01 (2.32e−03)+	9.15e+01 (2.25e−03)+	**4.06e+01** **(2.97e+01)**
RW- MOP2	1.37e+00 (2.83e−03)+	1.37e+00 (7.21e−04)+	1.37e+00 (1.81e−03)+	1.37e+00 (2.16e−03)+	1.34e+00 (2.52e−02)=	1.37e+00 (3.83e−04)+	1.37e+00 (3.13e−04)+	**1.34e+00** **(2.35e−02)**
RW- MOP3	6.06e+02 (1.59e−01)+	**6.05e+02** **(6.18e−02)−**	6.06e+02 (3.25e−01)+	6.05e+02 (5.67e−03)+	1.08e+03 (6.62e+02)+	6.05e+02 (2.52e−02)+	6.05e+02 (1.31e−02)+	6.41e+02 (1.95e+02)
RW- MOP4	1.55e+01 (2.79e−02)+	1.55e+01 (2.08e−02)+	1.55e+01 (2.36e−02)+	1.55e+01 (2.13e−02)+	1.51e+01 (3.12e−01)=	1.54e+01 (4.64e−02)+	1.55e+01 (2.30e−02)+	**1.36e+01** **(3.04e+00)**
RW- MOP5	3.72e−02 (1.41e-17)=	3.72e−02 (1.41e-17)=	3.72e−02 (1.41e-17)=	3.72e−02 (1.41e-17)=	3.72e−02 (1.41e-17)=	3.72e−02 (1.41e-17)=	3.72e−02 (1.41e-17)=	**3.72e−02** **(1.41e-17)**
RW- MOP6	3.05e+00 (8.73e−01)+	3.11e+00 (8.93e−01)+	6.31e+00 (2.40e+00)+	3.03e+00 (6.70e−01)+	3.20e+00 (8.77e−01)+	3.85e+00 (6.33e−01)+	3.26e+00 (7.32e−01)+	**9.02e−01** **(1.07e+00)**
RW- MOP7	1.21e−02 (3.53e-18)=	1.21e−02 (3.53e-18)=	1.21e−02 (3.53e-18)=	1.21e−02 (3.53e-18)=	1.21e−02 (3.53e-18)=	1.21e−02 (3.53e-18)=	1.21e−02 (3.53e-18)=	**1.21e−02** **(3.53e-18)**
RW- MOP8	9.29e+02 (1.17e+03)+	1.89e+02 (6.84e+01)+	2.89e+02 (8.22e+01)+	5.59e+02 (2.21e+00)+	5.85e+03 (5.10e+03)+	3.19e+02 (1.12e+02)+	3.10e+02 (1.22e+02)+	**3.29e+01** **(2.23e+01)**
RW- MOP9	1.61e−02 (1.16e−04)+	1.61e−02 (7.06e-18)+	1.61e−02 (7.06e-18)+	1.61e−02 (7.06e-18)+	1.58e+00 (1.38e−01)+	1.61e−02 (7.06e-18)+	1.61e−02 (7.06e-18)+	**1.61e−02** **(6.21e-18)**
RW- MOP10	NaN (0.00%)=	**1.31e+03** **(1.40e+02)−**	1.49e+03 (3.38e+02)−	NaN (0.00%)=	NaN (0.00%)=	1.49e+03 (1.89e+02)−	1.69e+03 (3.43e+02)−	NaN (0.00%)
RW- MOP11	7.44e−01 (2.18e−05)+	7.44e−01 (2.32e−05)+	7.44e−01 (8.50e−05)+	7.44e−01 (2.34e−05)+	7.44e−01 (2.99e−05)+	7.62e−01 (4.29e−02)+	7.44e−01 (2.13e−05)+	**7.44e−01** **(1.34e−05)**
RW- MOP12	2.60e−01 (1.25e−02)+	2.48e−01 (2.13e−03)=	**2.47e−01** **(2.73e−05)=**	2.77e−01 (2.16e−02)+	3.53e−01 (1.33e−02)+	2.48e−01 (2.54e−03)+	2.48e−01 (1.31e−03)=	2.48e−01 (1.37e−03)
+/−/=	9/0/3	7/2/3	8/1/3	9/0/3	7/0/5	9/1/2	8/1/3	

**Table 7:** HV results of the compared algorithms on the 12 real-world problems.

	BiCo	C3M	ToP	APSEA	CTAEA	DRCMO	MGCMOEA	IRCMO
RW- MOP1	**3.93e−01** **(1.95e−05)−**	3.93e−01 (1.68e−05)−	3.93e−01 (6.28e−04)−	3.68e−01 (9.28e−02)−	0.00e+00 (0.00e+00)+	3.93e−01 (1.30e−05)−	3.93e−01 (1.70e−05)−	2.59e−01 (9.67e−02)
RW- MOP2	8.59e−01 (1.66e−03)+	8.57e−01 (1.13e−03)+	**8.62e−01** **(1.66e−04)−**	8.55e−01 (3.98e−03)+	8.39e−01 (8.11e−03)+	8.57e−01 (1.66e−03)+	8.58e−01 (1.66e−03)+	8.60e−01 (1.76e−03)
RW- MOP3	**2.77e−01** **(9.26e−06)−**	2.76e−01 (1.27e−04)+	2.77e−01 (3.10e−04)−	2.77e−01 (4.01e−05)+	2.68e−01 (7.02e−03)+	2.77e−01 (2.78e−05)+	2.77e−01 (4.48e−05)+	2.77e−01 (4.64e−05)
RW- MOP4	4.85e−01 (2.59e−05)−	**4.85e−01** **(3.25e−05)−**	4.84e−01 (3.28e−05)+	4.85e−01 (3.28e−05)+	4.84e−01 (1.76e−04)+	4.85e−01 (3.06e−05)−	4.85e−01 (2.81e−05)−	4.85e−01 (3.83e−05)
RW- MOP5	**4.10e−01** **(3.04e−05)−**	4.09e−01 (1.13e−04)+	4.09e−01 (7.07e−05)+	4.09e−01 (9.28e−05)+	4.08e−01 (4.90e−04)+	4.09e−01 (5.99e−05)+	4.09e−01 (6.10e−05)+	4.10e−01 (3.75e−05)
RW- MOP6	5.56e−01 (1.37e−03)+	5.54e−01 (2.21e−03)+	**5.60e−01** **(1.55e−04)−**	5.53e−01 (3.62e−03)+	5.44e−01 (4.47e−03)+	5.54e−01 (1.88e−03)+	5.53e−01 (1.16e−03)+	5.58e−01 (8.52e−04)
RW- MOP7	6.17e−01 (5.99e−04)+	6.15e−01 (4.73e−04)+	6.18e−01 (8.90e−05)+	6.15e−01 (1.08e−03)+	6.16e−01 (1.29e−03)+	6.17e−01 (2.30e−04)+	6.17e−01 (1.76e−04)+	**6.18e−01** **(8.85e−05)**
RW- MOP8	5.43e−01 (8.46e−05)+	**5.44e−01** **(1.55e−05)−**	5.44e−01 (1.24e−05)−	5.43e−01 (1.38e−04)+	5.09e−01 (4.76e−02)+	5.43e−01 (1.30e−04)+	5.43e−01 (4.81e−05)+	5.44e−01 (9.97e−05)
RW- MOP9	3.18e−02 (3.31e−07)+	**3.18e−02** **(3.53e−07)−**	3.18e−02 (5.41e−07)+	3.18e−02 (5.83e−07)+	3.16e−02 (2.70e−05)+	3.18e−02 (4.95e−07)+	3.18e−02 (4.36e−07)+	3.18e−02 (5.94e−07)
RW- MOP10	NaN (0.00%)=	5.31e−01 (7.08e−02)−	**6.83e−01** **(2.11e−01)−**	NaN (0.00%)=	NaN (0.00%)=	5.18e−01 (1.41e−01)−	3.78e−01 (1.98e−01)−	NaN (0.00%)
RW- MOP11	2.42e−01 (7.12e−06)+	2.42e−01 (4.81e−06)+	2.41e−01 (9.44e−05)+	2.42e−01 (5.13e−06)+	2.41e−01 (1.77e−03)+	2.20e−01 (3.94e−02)=	2.42e−01 (6.19e−06)+	**2.42e−01** **(6.46e−06)**
RW- MOP12	1.35e−01 (4.83e−03)+	1.42e−01 (6.02e−03)−	**1.46e−01** **(8.73e−04)−**	1.26e−01 (7.88e−03)+	9.97e−02 (3.01e−03)+	5.09e−02 (8.10e−03)+	1.38e−01 (4.12e−03)=	1.38e−01 (3.74e−03)
+/−/=	7/4/1	6/6/0	5/7/0	10/1/1	11/0/1	8/3/1	8/3/1	

**Table 8:** Running time (s) of the compared algorithms on different problem sets. Each entry reports the mean value with the standard deviation in parentheses. The lowest mean runtime on each test set is highlighted in bold.

Test Set	BiCo	C3M	ToP	APSEA	C-TAEA	DRCMO	MGCMOEA	IRCMO
CF	**37.280544** **(2.492550)**	90.372503 (5.887471)	115.962004 (2.809446)	144.357588 (6.690925)	2021.506831 (80.156094)	540.873665 (29.817575)	512.348184 (29.568769)	323.671706 (14.967438)
DAS-CMOP	**55.658946** **(6.794478)**	253.152217 (19.635816)	523.511595 (11.427055)	83.868281 (23.737632)	1983.482374 (100.242442)	501.114548 (28.109251)	452.252668 (54.448199)	253.066660 (10.667027)
LIR-CMOP	**175.492841** **(10.881816)**	217.521428 (20.681600)	193.142643 (7.333112)	502.683880 (34.166340)	2785.395634 (60.439181)	1118.430369 (22.480715)	903.649965 (70.622666)	407.415404 (8.784939)
MW	121.972872 (3.486620)	**121.228608** **(7.616827)**	489.092988 (13.916140)	682.864020 (53.464661)	2975.997534 (98.802149)	743.918254 (37.006851)	815.640267 (64.213017)	554.699678 (8.740588)
RWMOP	790.303752 (132.534908)	1448.049777 (144.797745)	**102.604926** **(17.322561)**	3230.164250 (69.271921)	3612.400755 (98.171484)	1635.691577 (49.100254)	2305.202675 (55.269617)	642.535658 (11.578407)

**Table 9:** Wilcoxon signed-rank test results for IGD between IRCMO and the seven comparison algorithms over 59 problems.

IRCMO VS	$R^{+}$	$R^{-}$	*p*	Level = 0.05
BiCo	1470	70	$2.30329\times 10^{-9}$	YES
C3M	1204	392	$4.70331\times 10^{-4}$	YES
ToP	1386	210	$8.24454\times 10^{-7}$	YES
APSEA	1529	11	$1.0412\times 10^{-10}$	YES
C-TAEA	1389	207	$7.29546\times 10^{-7}$	YES
DRCMO	1413	183	$2.68597\times 10^{-7}$	YES
MGCMOEA	1465	131	$2.71387\times 10^{-8}$	YES

**Table 10:** Wilcoxon signed-rank test results for HV between IRCMO and the seven comparison algorithms over 59 problems.

IRCMO VS	$R^{+}$	$R^{-}$	*p*	Level = 0.05
BiCo	1657	54	$2.79423\times 10^{-10}$	YES
C3M	1334	436	$3.55545\times 10^{-4}$	YES
ToP	1637	133	$7.04449\times 10^{-9}$	YES
APSEA	1666	45	$1.7902\times 10^{-10}$	YES
C-TAEA	1497	214	$3.4734\times 10^{-7}$	YES
DRCMO	1525	245	$6.93267\times 10^{-7}$	YES
MGCMOEA	1590	180	$5.25872\times 10^{-8}$	YES

**Table 11:** Ablation settings for the core components of IRCMO.

Variant	Modification
IRCMO-A	Remove ITNES; replace auxiliary selection with constraint-agnostic nondominated sorting and crowding-distance selection while retaining the complete RCHRA.
IRCMO-B	Remove RCHRA; fix the offspring budgets of the two populations while retaining the complete ITNES.
IRCMO-C	Remove both ITNES and RCHRA, retaining only the basic dual-population framework.
IRCMO-D	Retain the squared excess-violation response and joint objective–decision niche exclusion of ITNES, but fix the tolerance threshold at τ=0 to assess the contribution of immune tolerance.
IRCMO-E	Retain reproductive success, homeostatic historical shares, geometric updating, and paired integer projection in RCHRA, but remove cross-population ecological complementarity to assess the contribution of complementarity.
IRCMO-F	Replace the squared excess-violation response in ITNES with a linear response.
IRCMO-G	Replace the joint objective–decision niche distance with objective-space distance only.

**Table 12:** Statistical results of IRCMO and its ablation variants on the 47 benchmark problems.

Algorithm	IGD	HV	IGD	HV
	(+/−/=)	(+/−/=)	Mean Rank	Mean Rank
IRCMO-A	39/4/4	40/4/3	3.9912	4.0646
IRCMO-B	46/0/1	47/0/0	4.9055	5.1257
IRCMO-C	41/2/4	43/2/2	4.9746	5.0678
IRCMO-D	46/1/0	46/1/0	5.9714	5.9055
IRCMO-E	46/0/1	47/0/0	4.7162	5.0230
IRCMO-F	46/0/1	47/0/0	5.3432	4.9789
IRCMO-G	46/0/1	47/0/0	4.9190	4.7269
IRCMO	\	\	1.1785	1.1071

**Table 13:** Sensitivity analysis of the minimum resource protection setting in RCHRA.

Parameter	IGD	HV	IGD	HV
Settings	(+/−/=)	(+/−/=)	Mean Rank	Mean Rank
$T_{\min}=\max\left\{1,0.05T\right\}$	46/0/1	47/0/0	3.3444	3.5361
$T_{\min}=\max\left\{1,0.15T\right\}$	46/0/1	47/0/0	3.5603	3.2357
$T_{\min}=\max\left\{1,0.35T\right\}$	46/0/1	47/0/0	3.4551	3.5750
$T_{\min}=\max\left\{1,0.45T\right\}$	46/0/1	47/0/0	3.6400	3.6531
$T_{\min}=\max\left\{1,0.25T\right\}$	\	\	1.0000	1.0000

## Data Availability

The data presented in this study are available on request from the corresponding author.
